# Novel Ellipsoid Chitosan-Phthalate Lecithin Nanoparticles for siRNA Delivery

**DOI:** 10.3389/fbioe.2021.695371

**Published:** 2021-07-28

**Authors:** Ramzi Mukred Saeed, Mohammed Abdullah, Mamoun Ahram, Mutasem Omar Taha

**Affiliations:** ^1^Department of Pharmaceutical Sciences, School of Pharmacy, The University of Jordan, Amman, Jordan; ^2^Department of Physiology and Biochemistry, School of Medicine, The University of Jordan, Amman, Jordan

**Keywords:** Chitosan nanoparticles, ellipsoid nanoparticles, non-viral vectors, siRNA delivery, gene delivery

## Abstract

Small interfering RNA (siRNA) has received increased interest as a gene therapeutic agent. However, instability and lack of safe, affordable, and effective carrier systems limit siRNA's widespread clinical use. To tackle this issue, synthetic vectors such as liposomes and polymeric nanoparticles have recently been extensively investigated. In this study, we exploited the advantages of reduced cytotoxicity and enhanced cellular penetration of chitosan-phthalate (CSP) together with the merits of lecithin (LC)-based nanoparticles (NPs) to create novel, ellipsoid, non-cytotoxic, tripolyphosphate (TPP)-crosslinked NPs capable of delivering siRNA efficiently. The resulting NPs were characterized by dynamic light scattering (DLS) and transmission electron microscopy (TEM), and were found to be ellipsoid in the shape of *ca*. 180 nm in size, exhibiting novel double-layer shells, with excellent stability at physiological pH and in serum solutions. MTT assay and confocal fluorescence microscopy showed that CSP-LC-TPP NPs are non-cytotoxic and efficiently penetrate cancer cells *in vitro*. They achieved 44% silencing against SLUG protein in MDA-MB-453 cancer cells and were significantly superior to a commercial liposome-based transfection agent that achieved only 30% silencing under comparable conditions. Moreover, the NPs protected their siRNA cargos in 50% serum and from being displaced by variable concentrations of heparin. In fact, CSP-LC-TPP NPs achieved 26% transfection efficiency in serum containing cell culture media. Real-time wide-field fluorescence microscopy showed siRNA-loaded CSP-LC-TPP NPs to successfully release their cargo intracellularly. We found that the amphoteric nature of chitosan-phthalate polymer promotes the endosomal escape of siRNA and improves the silencing efficiency.

## Graphical Abstract

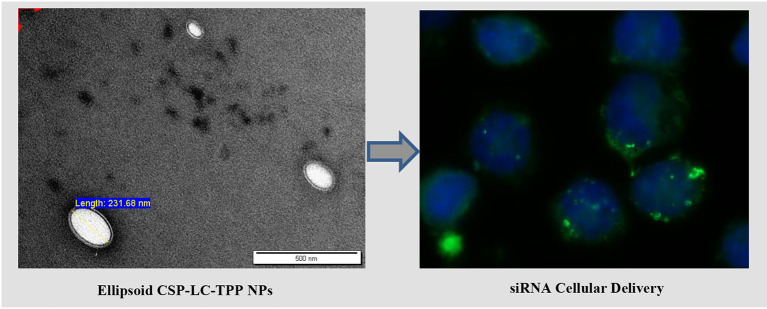

## Introduction

Gene therapy is a promising treatment approach for genetic diseases. It proceeds by the introduction of a corrected gene(s) or silencing the gene(s) responsible for overexpression of specific pathogenic protein(s) (Shahryari et al., 2019). One particular means of gene silencing is the delivery of small-interfering RNA (siRNA) into cells. siRNA has received increased interest as a gene therapeutic agent in a variety of diseases because of its specificity and versatility (Hu et al., 2020). However, siRNA is unstable in biological fluids, e.g., serum, because of the abundance of endonucleases (Whitehead et al., 2009). Moreover, siRNA should cross several biological barriers before reaching its site of action, i.e., the RNA-induced silencing complex (RISC) in the cellular cytoplasm (Kim et al., 2019). These issues greatly undermine the clinical advancement of siRNA (Nguyen and Szoka, 2012) and necessitate the need for effective carrier systems for siRNA delivery.

Still, the lack of safe, affordable, and effective carrier systems for delivering siRNA is limiting the widespread use of siRNA for gene therapy (Shukla et al., 2019).

To overcome these hurdles, extensive research has been done on synthetic vectors development, such as liposomes, polymeric nanoparticles, protein nanoparticles, and dendrimers (Abozeid et al., 2016; Pinnapireddy et al., 2019; Tariq et al., 2019; Bono et al., 2020; Chen et al., 2020). Synthetic delivery vectors offer many advantages over viral vectors, such as evading the anti-viral immune response, excellent safety profile, simple construction, low production costs, and the ability to insert a large-sized gene in the carrier system (Lostalé-Seijo and Montenegro, 2018).

Ideal carrier intended to deliver DNA or siRNA to the site of therapy must accomplish five tasks: (1) pack nucleic acids into a nanocarrier system; (2) protect the cargo and direct it specifically into diseased cells; (3) facilitate the endosomal escape of nucleic acids; (4) release of nucleic acid cargo into the cytoplasm; (5) get metabolized into non-toxic fragments; and, most importantly, (6) prevent the elicitation of immune responses (Lostalé-Seijo and Montenegro, 2018; Kim et al., 2019; Wahane et al., 2020).

**Figure 1:**
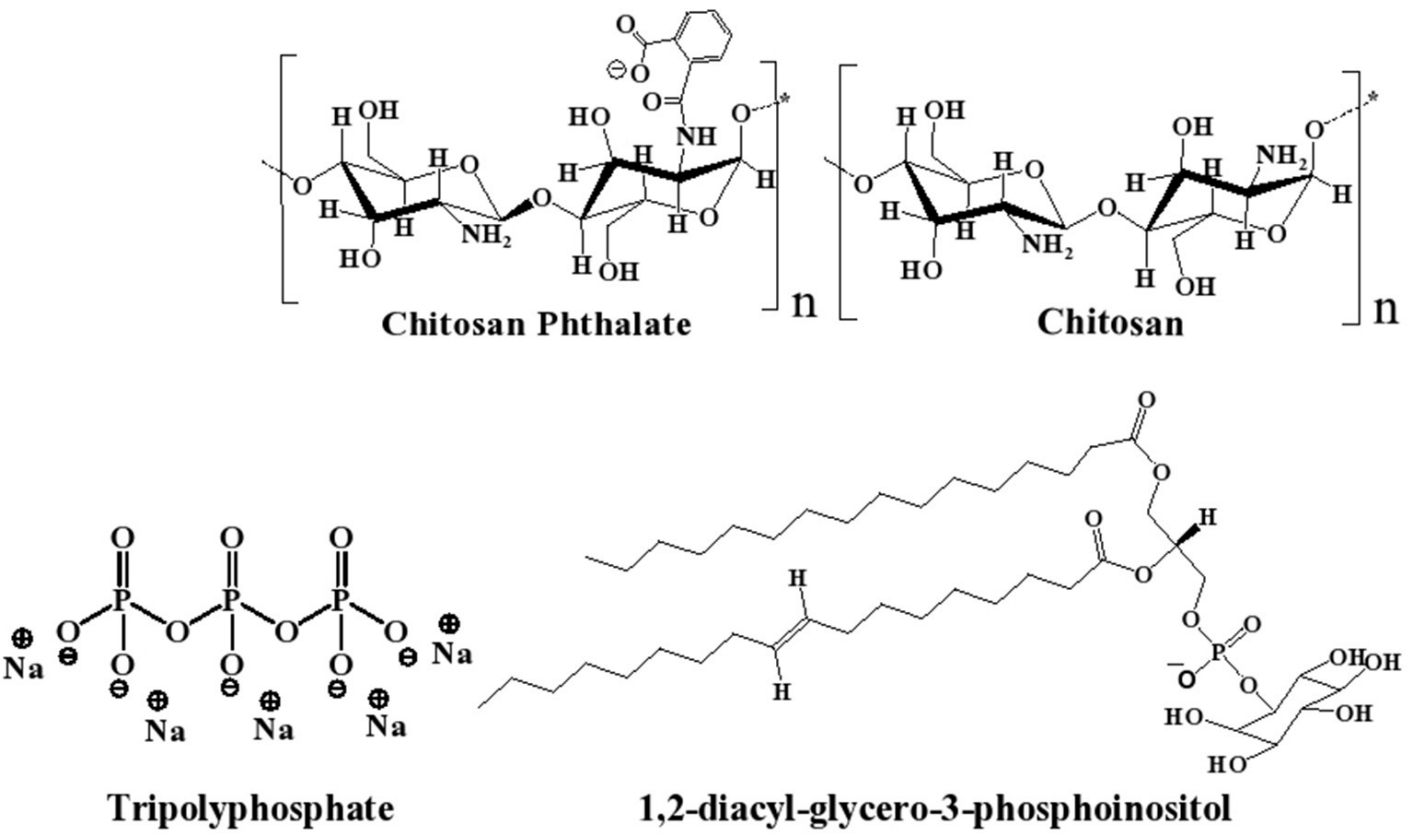
Chemical structures of chitosan (CS), chitosan-phthalate (CSP), diacylphospho-inositol (major component of lecithin), and tripolyphosphate (TPP).

Polymer-based siRNA delivery systems, e.g., chitosan (CS, Figure 1), have attracted attention (Sharma et al., 2019). CS is a well-known biocompatible natural polymer of low immunogenic properties. It exhibits additional advantages of muco-adhesiveness, cell permeation enhancement properties, and promotion of siRNA endosomal escape (Mansouri et al., 2004; Sarmento et al., 2011; Pellá et al., 2020). CS-based nanoparticles (NPs) have been studied previously as non-viral gene delivery tool, because they provide good packing capacity for DNA and siRNA (Farid et al., 2014; Baghdan et al., 2018; Böker et al., 2019; Cao et al., 2019; Ziminska et al., 2020). However, the cationic nature of CS causes stability and cytotoxicity issues (Rai et al., 2019; Saeed et al., 2020), and, therefore, limits the realization of the full potential of CS NPs as clinically viable siRNA delivery tool (Kargaard et al., 2019; Thomas et al., 2019).

Lipid-based nanocarriers have gained great significance as therapeutic delivery systems for siRNA in the last two decades (Li et al., 2020). Lecithin (LC) is a safe food additive that occurs naturally as a mixture of diglycerides of stearic, palmitic, and oleic acids, linked to the choline ester of phosphoric acid, as in Figure 1 (Fiume, 2001). Polymer-lipid hybrid carriers combine the merits of liposomes and polymers to create a nanoscale, biocompatible, and efficient delivery system for genetic materials (Schäfer et al., 2010; De Jesus and Zuhorn, 2015; Pinnapireddy et al., 2019). Hybrid lipid core/CS shell nanoparticles have been studied for drug (Ridolfi et al., 2012; Ramasamy et al., 2014), protein (Sarmento et al., 2011), and nucleic acid delivery (Danhier et al., 2015).

LC/CS-based NPs have been reported as successful *in vitro* and *in vivo* drug delivery vehicles (Sonvico et al., 2006; Chadha et al., 2012; Correa et al., 2020; Murthy et al., 2020). Moreover, CS-lecithin (LC, Figure 1) nanocomplex was also reported to be useful for the protection of siRNA in serum and subsequent cellular delivery (Sarmento et al., 2011; Trickler et al., 2011; Delgado et al., 2013). For example, LC/CS/protamine/thiamine pyrophosphate-based NPs were reported to be stable and successfully protected and delivered siRNA cargo in serum conditions (Ki et al., 2014). However, the LC content of such NPs is significantly higher than CS content (at least double the amount), which renders them susceptible for quick systematic clearance (Robinson, 1996; Tezgel et al., 2018; Besin et al., 2019) and prompt degradation at 37°C (Robinson, 1996; Tezgel et al., 2018; Besin et al., 2019).

As part of the quest of the authors to develop stable, efficient, and non-toxic NPs for drug delivery (Abulateefeh and Taha, 2015; Dmour and Taha, 2017; Saeed et al., 2020), we became interested in developing novel LC-stabilized CS NPs for delivering siRNA. In this study, we envisaged a hybrid NPs system of the non-cytotoxic CS derivative, i.e., CSP (Dmour and Taha, 2017; Saeed et al., 2020) combined with lesser amounts of LC, i.e., compared with published systems ( ≤ 20% w/w). The system is intended to combine the advantages of LC for NP stabilization and biocompatibility (Delgado et al., 2013; Ki et al., 2014) with gene packing of CS and cellular penetration capabilities (Sarmento et al., 2011; Baghdan et al., 2018; Cao et al., 2019; Saeed et al., 2020). TPP is added to provide additional stability to NP formulation and reduce the amounts of LC required to promote NP formation and improve NP size properties by ionotropically crosslinking the CS (or CSP) content. The proposed NPs are anticipated to have TPP-crosslinked CSP-based outer shell and inner LC-based core (see Figure 1 for chemical structures). This is the first time that the use of CS/LC or CSP/LC with TPP crosslinker to generate NPs for gene delivery has been reported.

The resulting NPs were characterized by DLS, TEM, and fluorescence microscopy, and were found to efficiently deliver siRNA into breast cancer cells and silence the oncogenic SLUG protein. We compared the performance of NPs based on unmodified CS with that of CSP upon the combination with LC and TPP. Interestingly, CSP-LC-TPP NPs were superior, and in fact, they significantly superseded the commercial transfection agent, RNAifectin. Additionally, they were found to be on par with covalently stabilized NPs (Abozeid et al., 2016) *vis-à-vis* exceptional stability under physiologically relevant conditions. Moreover, our NPs are self-assembled without the need for potentially toxic covalent crosslinkers (e.g., glutaraldehyde) that require extensive washing prior to administration (Fürst and Banerjee, 2005; Islam et al., 2019).

SLUG is a zinc-finger transcription factor that belongs to a larger superfamily known as SNAI and participates in cell differentiation, survival, and epithelial-mesenchymal transition (EMT) (Ganesan et al., 2016). Activated Notch 1 signaling induces EMT and promotes invasion and metastasis of breast cancer cells through overexpression of SLUG (Cao et al., 2015). SLUG was reported to be expressed in MDA-MB-453 cells (Martin et al., 2005) and to be critical for the progression of invasive breast ductal carcinoma (Carpenter et al., 2015).

## Materials and Methods

### Materials

All chemicals were purchased from respective companies and were used without pretreatment or purification. siRNA for SLUG mRNA (sense sequence: CAAUAAGACCUAUUCAACUtt, antisense sequence: AGUUGAAUAGGUCUUAUUGca) was purchased from Ambion (Elk Grove, CA, United States). RNAifectin was purchased from Applied Biological Materials Inc. (British Columbia, Canada). Amicon Ultra Centrifugal Filter Units were obtained from Millipore (Darmstadt, Germany). Soya bean lecithin was purchased from Sigma-Aldrich (Darmstadt, Germany). Absolute ethanol, pyridine, and acetone of analytical grades were purchased from Carlo Erba (Val-de-Reuil, France) and Labchem (Pittsburgh, PA, United States). Medium molecular weight chitosan was obtained from Sigma-Aldrich (St. Louis, MI, United States). Phthalic anhydride was purchased from Fluka (Switzerland). Ultrapure water for DLS studies with 0.05 μs/cm conductivity was obtained from Millipore (Burlington, MA, United States). RNase-free water was obtained from Qiagen (Hilden, Germany). Penta basic sodium tripolyphosphate was purchased from Sigma-Aldrich (Darmstadt, Germany). Hydrochloric acid (37%) was purchased from Carlo Erba (Barcelona, Spain) and sodium hydroxide was purchased from Rasayan Laboratory (Gujarat, India). Doxorubicin (DOX) was obtained from Ebewe Pharma (Attersee, Austria). FBS was purchased from Biowest (Nuaille, France). Leibovitz's L-15 medium, L-glutamine, penicillin–streptomycin, and trypsin-EDTA were purchased from Capricorn Scientific (Ebsdorfergrund, Germany). ProLong Diamond Antifade Mountant 4′,6-Diamidino-2-phenylindole (DAPI) stain and UltraPure Agarose were obtained from Invitrogen (Waltham, MA, United States) and ThermoFisher Scientific (Waltham, MA, United States). Poly-L-lysine and heparin sulfate were purchased from Sigma-Aldrich (Darmstadt, Germany). Human breast MDA-MB-453 cancer cell line was purchased from American Type Culture Collection (ATCC, Manassas, VA, United States). SiRNA AF 488 was purchased from Qiagen (Evois, Finland). The RedSafe dye was obtained from Intron Biotechnology (Gyeonggi, South Korea). TBE buffer was purchased from Biotech (Ontario, Canada).

### Preparation of NPs

Chitosan-phthalate (CSP) was prepared as described earlier (Dmour and Taha, 2017; Saeed et al., 2020). CS-LC, CSP-LC, and their corresponding TPP-crosslinked NPs were prepared by simple mixing. CS, or CSP was dissolved in aqueous HCl (4.8 mM) to produce 0.1% w/v solution by stirring over 24 h. The resulting solution was filtered and centrifuged at 4,000 rpm for 10 min at 25°C to remove any insoluble polymeric residues. The pH of the polymeric solutions was adjusted by the dropwise addition of NaOH (1 M) to 5.5 or 4.6 for CS or CSP solutions, respectively. Subsequently, ethanolic solution of LC (volumes are shown in Table 1, 1% w/v) with or without freshly prepared TPP aqueous solution (volumes are shown in Table 1, 0.1% w/v) was added dropwise to the prepared CS and CSP solutions (2.5 ml) under vigorous magnetic stirring at 25°C until visual appearance of hazy opalescent dispersion representing NPs formation. The NP dispersion was left under stirring for over 1 min. The resulting NPs were used for stability evaluation, and size and zeta potential determination (i.e., dynamic light scattering, DLS).

**Table 1:** Prepared NPs, their abbreviations and corresponding amounts of components required for formulation.

**NPs names**	**NPs abbreviations**	**Volumes (ml)**			
		**Chitosan**	**Lecithin**	**Chitosan-to-Lecithin**	**TPP**
		**(0.1%w/v)**	**(1.0%w/v)**	**ratio**	**(0.1% w/v)**
Chitosan-lecithin	CS-LC	2.5	0.20	1.25:1	–
Chitosan-phthalate-lecithin	CSP-LC	2.5	0.20	1.25:1	–
Chitosan-lecithin-tripolyphosphate	CS-LC-TPP	2.5	0.05	5:1	0.315
Chitosan-phthalate-lecithin-tripolyphosphate	CSP-LC-TPP	2.5	0.05	5:1	0.087

However, for doxorubicin (DOX)- or siRNA-loaded NPs intended for cellular uptake or transfection studies, DOX (0.25 mg), or siRNA (30 μl, 10 μM) was magnetically stirred with the polymeric solution (CS or CSP, 2.5 ml, 0.1% w/v) for 5 min. Subsequently, LC or LC-TPP mixture (volumes and concentrations are shown in Table 1) was added dropwise to the polymeric solution until the visual appearance of hazy opalescent dispersion representing NPs formation. The formulated NPs were left over 5 min on the shelf before further processing. Subsequently, the NP mixture suspensions (*ca*. 2.5 ml) were concentrated to 0.6 ml using Amicon Ultra Centrifugal Filter Units (100 KDa, Millipore, Darmstadt, Germany) and centrifugation for over 10 min at 10°C and 6,000 RPM. Thereafter, the NPs were washed using phosphate buffer (1 ml) followed by centrifugation at the same conditions to concentrate the NPs (0.6 ml). Washing was performed two times to remove excess unloaded siRNA. Aliquots of resulting NPs suspensions (200 μl) were added over attached cells (see sections Transfection studies and Assessment of NP cellular uptake by fluorescence microscopy) to reach final concentrations of 100 and 650 nM for siRNA and DOX per well, respectively. A similar procedure was performed to prepare blank NPs and for cytotoxicity experiments, albeit without adding cargo (siRNA or DOX). All aqueous solutions for the preparation of NPs for cellular uptake, transfection, and cytotoxicity studies were prepared using nuclease-free water (Qiagen, Hilden, Germany).

### Encapsulation Efficiency

siRNA loading within NP formulations was studied as previously reported (Farid et al., 2014). Briefly, siRNA-loaded NP formulations were prepared as mentioned above (section Preparation of NPs). The resulting NPs dispersions (ca. 2.5 ml) were collected and concentrated to 0.6 ml using Amicon Ultra Centrifugal Filter Units (100 KDa, 4 ml, Millipore, Darmstadt, Germany) by centrifugation for 10 min at 10°C and 6,000 RPM. The free-from-NP filtrates were collected in eppendorf tubes and their amounts of siRNA were measured using BioDropμLITE Spectrophotometer (ThermoFisher Scientific, Waltham, MA, United States). The filtrate of unloaded NPs was used as blank. The percentage of siRNA entrapped in certain NP formulation was determined using the following equation:

$$\%Loading=\frac{Total\;amounts\;of\;added\;siRNA-Amount\;of\;nonentrapped\;siRNA\;in\;Filtrate}{Total\;amounts\;of\;added\;siRNA}$$

### Agarose Gel Electrophoresis

#### Stability of siRNA-Loaded NPs in FBS

The stability of loaded NPs against serum degradation was studied by gel electrophoresis. NPs samples suspended in PBS (pH 6.8 or 7.4, 15 μl), each containing siRNA (0.4 μg), were gently vortexed with the FBS solution (100%, 15 μl, at pH 6.8 or 7.4) and incubated at 37°C for 1 h. Subsequently, the resulting dispersion (25 μl) was mixed with 6X Gel Blue Loading Dye (5 μl) and loaded onto 2% agarose gel. Naked siRNA and siRNA-loaded NPs at preparation pH were used as controls. The electrophoresis was carried out at a constant voltage of 100 V for 40 min in Tris-Borate-EDTA (TBE) buffer containing 1X RedSafe dye (0.05 μl/ml) and visualized under a UV trans-illuminator at a wavelength of 254 nm (Bio-Rad, Marnes-la-Coquette, France).

#### Heparin-siRNA Competition Assay

Heparin-siRNA competition assay was studied as previously reported (Malfanti et al., 2019). Briefly, siRNA-loaded NPs samples (20 μl, loaded with 0.4 μg siRNA) were prepared and resuspended in PBS at pH 6.8 or 7.4. The NPs were then added to RNase-free PBS or heparin solutions (5 μl) in RNase-free PBS adjusted at target pH (6.8 or 7.4) to yield heparin concentrations in the range of 0–15 IU/30 μl in the final NPs dispersion. The samples were incubated at 37°C for 3 h, with the loading dye, and were finally analyzed by gel electrophoresis according to the procedure reported in section (Stabilities ofsiRNA-Loaded NPs in FBS).

### NPs Characterization

#### Dynamic Light Scattering Analysis of NPs Under Variable pH and 10% FBS Conditions

Aliquots of NPs dispersions (2 ml) were evaluated by dynamic light scattering (DLS) either directly (at preparation pH), after 2 h of exposure to variable pH conditions (1.2, 6.8, and 7.4) or to fetal bovine serum [FBS, 10% w/v in phosphate buffer solution (PBS) at pH 7.4]. pH adjustments were achieved by aqueous NaOH or HCl (1M). Only samples with hazy appearance were investigated by DLS, while those showing aggregates were discarded. The samples were evaluated by DLS after 2 h of exposure to variable pH or 10% FBS solutions. Nanoparticle size, zeta potential, and polydispersity index (PDI) were determined by measuring the electrophoretic mobility of NP dispersion and using the Stokes–Einstein and Henry equations (Media viscosity = 0.8872 cP, dielectric constant = 78.5, temperature = 25°C). The calculations were performed using the Zetasizer software (version 7.11). The average size and zeta potential of triplicate measurements at 25°C were recorded. The shape characteristics of NPs were studied by TEM [Morgagni (TM) FEI 268, Holland] using Mega-View Camera. The NP samples were immobilized on copper grids for 10 min and dried at room temperature before investigation by TEM.

#### Transfection Studies

MDA-MB-453 cells were cultivated and maintained in Leibovitz's medium supplemented with 10% FBS, 1% L-glutamine, and 1% penicillin/streptomycin at 37°C. This combination is termed as a complete medium. Cells were trypsinized by trypsin-EDTA and centrifuged to form pellets. The supernatant was discarded. The cell pellets were then resuspended in the culture medium. The stock cell suspension was diluted to the required concentration (15 ×10^4^ cells/well) in culture medium and transferred to 6-well-plates by adding 1,000 μl to each well. The plates were incubated in a humidified atmosphere at 37°C for 24 h to allow cell attachment. Amounts of NPs to give a final concentration of 100 nM siRNA per well were added to each well and incubated for 5 h with or without 10% FBS. Subsequently, the media in each well were replaced by completely fresh media. The cells were then kept for 72 h and collected for protein assay analysis. Cells treated with blank NPs were used as negative controls. Cells treated with RNAifectin loaded with siRNA were used as positive controls and prepared as follows: RNAifectin (5 μl) was diluted in 125 μl of the serum-reduced medium, and siRNA (10 μl, 10 μM) was diluted in 125 μl of the serum-reduced medium. Both RNAifectin and siRNA solutions were mixed and incubated at room temperature for 20 min. The mixture was then applied onto the cultured cells to get a final concentration of 100 nM siRNA per well and incubated for 5 h. Subsequently; the media were replaced with complete fresh media and incubated for 72 h.

#### Western Blotting

Protein extraction was performed as previously reported (Ahram et al., 2018). RIPA-lysis buffer (ThermoFisher Scientific, Waltham, MA, United States) was used to disrupt the cellular cytoplasm 72 h post-treatment. The lysates were agitated at 4°C for 30 min and then centrifuged at 720 RPM for 5 min. The supernatant containing the protein fraction was used for analysis. Sodium dodecyl sulfate-polyacrylamide gel electrophoresis (SDS-PAGE) was used to analyze SLUG protein expression. Gels were prepared as 12.5% acrylamide-bisacrylamide. The extracted proteins were transported onto a nitrocellulose membrane of 0.45-μm pore size (Santa Cruz). The following antibodies were used in the analysis: mouse anti-SLUG antibody (clone A-7, 1:500; Santa Cruz, United States), mouse anti-GAPDH (ab8245;, 1:5,000, Abcam, Cambridge, United Kingdom), and secondary goat anti-mouse antibody (ab97023; Abcam, Cambridge, United Kingdom). Densitometric analysis was performed on the blots using Image lab software version 6.1 (Bio-Rad) and GraphPad Prism version 8.0.2 for data analysis.

### Assessment of NPs Cellular Uptake by Fluorescence Microscopy

#### Cell Uptake of DOX-Loaded NPs

NP cell uptake was studied as previously described (Saeed et al., 2020) with slight modifications. Human breast MDA-MB-453 cancer cells were seeded onto collagen-coated round cover slips (prepared by incubation with 0.01% w/v aqueous solution of Type 1 collagen from rat-tail (Sigma-Aldrich, St. Louis, MI, United States) for 1 h at 25°C) in a 12-well-plate at 5 × 10^−4^ cells/well in culture medium (Leibovitz's medium supplemented with 10% fetal bovine serum, 1% L-glutamine, and 1% penicillin/streptomycin) and left for 24 h. Subsequently, suspended DOX-loaded NPs (0.25 mg) or the free DOX solution (175 μl, 2 mg/ml to yield a final concentration of 650 nM) in tissue culture media was directly applied to coverslips-adhered cells and incubated for 4 h at 37°C. Consequently, the culture media were discarded, and the wells were washed two times with PBS. The cells were then fixed by 4% paraformaldehyde solution (at room temperature for over 20 min then washed two times with 1 ml PBS). Subsequently, triton-x solution (0.5% v/v) was added to the wells and kept for 10 min then washed two times with PBS (1 ml). After that, the coverslips were detached and slowly flipped over clean glass slides coated with 50 μl DAPI stain (Prolong™ Diamond AntifadeMountant with DAPI) and kept overnight at 25°C and under dark conditions. Fixed cells were imaged at laser/detector wavelengths of 488 nm/614–742 nm and 405 nm/410–585 nm for DOX and DAPI, respectively, *via* a confocal laser scanning microscope (LSM 780, Carl Zeiss, Oberkochen, Germany) with 63 × /1.4 oil lens. Untreated cells (i.e., with DOX-loaded NPs or free DOX) were evaluated as controls. NP uptake was also studied *via* wide-field fluorescence microscopy (Axio Imager Z2, Carl Zeiss, Oberkochen, Germany).

#### Cell Uptake of siRNA-Loaded NPs

NPs (CSP-LC-TPP and CS-LC-TPP) loaded with siRNA (AF488, fluorescent scrambled siRNA) were prepared as mentioned in section (Preparation of NPs). Then, volumes of NP suspensions equivalent to 50 nM siRNA AF488 were incubated for 4 h with MDA-MB-453 cancer cells. The cells were then fixed on coverslips as mentioned above, and the coverslips were imaged at laser/detector wavelengths of 488 nm/500–530 nm *via* a confocal laser scanning microscope (63 × /1.4 oil lens, LSM 780, Carl Zeiss, Oberkochen, Germany). MDA-MB-453 cancer cells incubated over the same time interval (4 h) with equivalent amounts of loaded RNAifectin or naked siRNA (50 nM siRNA AF488 each) imaged as positive or negative controls, respectively.

#### NPs Cell Uptake and Intracellular Release of siRNA Cargo Over Time

Wide-field fluorescence microscopy was performed to assess siRNA-loaded NP cell uptake and siRNA release over 2, 4, and 6 h as reported previously (He et al., 2019). Briefly, NPs (CSP-LC-TPP and CS-LC-TPP) loaded with siRNA (AF488, fluorescent scrambled siRNA) were prepared as mentioned in section Preparation of NPs. Then, volumes of NP suspensions equivalent to 50 nM siRNA AF488 were incubated for 2, 4, or 6 h with MDA-MB-453 cancer cells. The cells were then fixed on coverslips as mentioned above, and the coverslips were imaged at wavelengths of 488 nm/500–530 nm *via* a wide-field fluorescence microscope (100 × /1.4 oil lens, observer. Z1, Carl Zeiss, Oberkochen, Germany).

#### Cytotoxicity Study

MDA-MB-453 cells were cultivated and maintained in complete culture media at 37°C. The cells were trypsinized by trypsin-EDTA and centrifuged to form pellets. The pellets of cells were re-dispersed in a complete medium and diluted to the desired concentration (15 × 10^4^ cells/ml) then transferred to 6-well-plates by adding 1 ml to each well. The plates were incubated in a humidified atmosphere at 37°C for 24 h to allow cell attachment at the time of NP treatment. Then, the culture media were discarded and replaced by fresh media with an appropriate amount of NP suspension or RNAifectin. After incubation for 72 h, the MTT solution was added into each well to a final concentration of 5 mg/ml and incubated at 37°C for an additional 3 h, and then the media were discarded and replaced with DMSO/isopropanol solution (200 μl, 50% v/v) into each well. Thirty minutes later, cellular viability was determined by measuring the absorbance at 570 nm using Synergy HTX Multi-Mode Plate Reader (BioTek Instruments, Winooski, VT United States). The results of the MTT proliferation assay were analyzed using Microsoft Excel. The cellular morphological changes related to RNAifectin or NPs-induced cytotoxicity were monitored using an inverted microscope (DMIL LED, Leica Microsystems, Wetzlar, Germany). Untreated cells were used as controls.

## Results and Discussion

### NPs Formulation, Sizes, Surface Charges, and Behavior Under Variable pH Conditions and 10% FBS

The new NPs were prepared by mixing CS or CSP with LC in the presence or absence of TPP ionotropic crosslinker. Four NP formulations were generated, namely, CS-LC, CSP-LC, CS-LC-TPP, and CSP-LC-TPP NPs (see Table 1). Ionotropic gelation proceeds *via* electrostatic interaction between protonated amine groups of CS or CSP with phosphate anions of LC and/or TPP.

We started by preparing CS-LC or CSP-LC NPs without TPP. Upon testing several CS/LC and CSP/LC combinations, we concluded that the maximum CS-to-LC or CSP-to-LC ratios that allow viable NP formation was 1.25:1 (w/w). This was achieved by setting the preparation pH to 5.5 and 4.6 for CS and CSP, respectively. However, it was possible to increase CS (or CSP) content to 5:1 (compared with LC, Table 1) upon adding TPP to the NP formula. Although this ratio has not been reported for CS-LC NPs, it is still not unexpected since TPP efficiently crosslinks CS (or CSP) and reduces reliance on LC for NP formation. NP formation was confirmed by the advent of hazy dispersion and by dynamic light scattering. NP sizes and surface charges are shown in Tables 2, 3.

**Table 2:** Size properties of unloaded-NPs under varying pH and 10% FBS solution.

**NPs**	**NPs property^***a***^**	**At preparation conditions**	**pH**			
			**1.2**	**6.8**	**7.4**	
					**Aqueous conditions**	**FBS (10% v/v)**
CS-LC	Size (nm)	375.5 ± 7.9^b^	281.5 ± 7.4	607.1 ± 20.2	3,222 ± 194	Aggregate
	PDI	0.55 ± 0.04	0.38 ± 0.04	0.72 ± 0.14	0.47 ± 0.33	–
CS-LC-TPP	Size (nm)	270.5 ± 17.9^c^	205.2 ± 3.5	220.9 ± 12.9	239.5 ± 18.6	179.1 ± 13.3
	PDI^b^	0.47 ± 0.03	0.29 ± 0.01	0.45 ± 0.03	0.49 ± 0.09	0.50 ± 0.02
CSP-LC	Size (nm)	364.9 ± 28.3^d^	207.0 ± 7.4	294.5 ± 12.8	6,791 ± 646	Aggregate
	PDI^b^	0.73 ± 0.13	0.53 ± 0.06	0.287 ± 0.02	1.00 ± 0.00	–
CSP-LC-TPP	Size (nm)	171.2 ± 13.8^e^	178.1 ± 9.5	157.6 ± 19.0	186.2 ± 9.4	243.9 ± 19.5
	PDI^b^	0.318 ± 0.05	0.253 ± 0.01	0.22 ± 0.01	0.288 ± 0.03	0.414 ± 0.11

a *Each value represents the average of triplicate measurements ± standard deviation.*

b *Preparation pH is 5.70,*

c *Preparation pH is 6.10,*

d *Preparation pH is 4.85,*

e *Preparation pH is 5.42*.

**Table 3:** Unloaded-NPs zeta potentials at varying pH conditions.

	**Zeta potential (mV)** ^***a***^				
**NPs**	**Preparation pH**	**pH 1.2**	**pH 6.8**	**pH 7.4**	
				**Aqueous conditions**	**FBS (10% v/v)**
CS-LC	+43.3 ± 7.9	+20.0 ± 2.9	+22.0 ± 2.3	+12.9 ± 4.4	Aggregate
CS-LC-TPP	+32.5 ± 1.16	+21.4 ± 3.5	+4.9 ± 1.1	+4.3 ± 1.1	+3.0 ± 0.6
CSP-LC	+38.2 ± 3.2	+18.4 ± 2.8	+12.8 ± 1.4	+2.3 ± 1.9	Aggregate
CSP-LC-TPP	+30.1 ± 1.2	+19.3 ± 2.4	+3.1 ± 1.1	−7.5 ± 0.4	−3.0 ± 0.4

a *Each value represents the average of triplicate measurements ± standard deviation. Supplementary Figure 1 shows examples of DLS graphs with run parameters*.

Noticeably, all prepared NPs (unloaded) exhibited significant cationic surface charges, as shown in Table 3, even under variable pH conditions (except for CSP-LC-TPP, which became slightly negative at pH 7.4, Table 3), indicating that their outer shells were composed mainly of cationic CS or CSP, while negatively charged LC remained mainly within NP cores. This conclusion is in parallel with previous reports (Chu et al., 2019; Jardim et al., 2020; Murthy et al., 2020).

Predictably, TPP-crosslinked NPs were smaller by at least 100nm (sizes ≤ 270nm) compared with their TPP-deprived counterparts under comparable pH conditions (Table 2). Moreover, TPP conferred significant stability to CS-LC-TPP and CSP-LC-TPP NPs, particularly under physiological pH conditions and in 10% FBS (in PBS). On the other hand, although CS-LC and CSP-LC NPs were stable under acidic and pH 6.8 conditions, they became micro-sized at physiological pH and aggregated in 10% FBS. Needless to say, serum lipases, nucleases, and high-density lipoproteins promote the disruption of lipid-based NPs (Rao, 2010).

TPP stabilizes CS-LC and CSP-LC NPs probably by electrostatic attraction with slack cationic chitosan shell layers and thus compacts them, causing NPs size to collapse and enhance NPs stability. This assumption correlates with the observation that TPP-based NPs (CS-LC-TPP and CSP-LC-TPP) exhibit reduced cationic surface charges compared with their TPP-deprived counterparts (CS-LC and CSP-LC, Table 3), indicating that TPP ions deposit at NP shells and neutralize their positive surface charges as previously reported (Saeed et al., 2020). Nevertheless, CSP-LC-TPP NPs score the least surface positive charge (Table 3) and smallest size (average size of 180 nm, Table 2) of all the NP formulas and under variable pH conditions. In fact, under physiological pH conditions and 10% FBS, CSP-LC-TPP NPs exhibited negative charges, probably because of the deprotonation of grafted phthalate residues of CSP. The collective effects of negative charges from TPP and grafted phthalate should cause stronger electrostatic crosslinking of surface CSP NPs, leading to the observed small sizes and better NP stability.

Table 4 shows how siRNA loading affected NP properties and the encapsulation efficiency of different NP formulations. Interestingly, all the NP formulas showed excellent encapsulation capacities presumably because of the electrostatic attraction between CS or CSP with siRNA polyanion during NP formation. This finding is in agreement with previous studies that have reported up to 100% encapsulation efficiency of siRNA by chitosan-based NP formulations (Ragelle et al., 2014; Alameh et al., 2018). Interestingly, siRNA loading was accompanied by negligible changes in the surrounding pH, which should preserve the electrostatic characters of the attracting parties, i.e., siRNA polyanion and polymers (CS or CSP), and, thus, enhance NP encapsulation capacities.

**Table 4:** Sizes and positive charge of siRNA-loaded NPs and the encapsulation efficiencies.

**NPs**	**At preparation pH**			**At pH 7.4**		
	**Size (nm)^**a**^, ^**b**^**	**PDI^**a**^, ^**b**^**	**Zeta potential (mV)^**a**^, ^**b**^**	**Size^**b**^**	**PDI^**b**^**	**Zeta potential (mV)^**b**^**	**EE^**b**^, ^**c**^**
CS-LC	128.6 ± 6.13	0.59 ± 0.12	+43.0 ± 1.4	ND	ND	ND	99 ± 0.52%
CS-LC-TPP	192.7 ± 4.30^d^	0.39 ± 0.04	+19.0 ± 1.0	345.5 ± 6.05	0.202 ± 0.03	−5.93 ± 1.02	99 ± 0.23%
CSP-LC	93.99 ± 2.06	0.40 ± 0.01	+30.3 ± 0.5	ND	ND	ND	98 ± 0.14%
CSP-LC-TPP	211.6 ± 2.61^e^	0.22 ± 0.01	+29.1 ± 0.5	254.5 ± 4.56	0.35 ± 0.001	−8.27 ± 0.91	98 ± 0.68%

a *Determined at preparation pH*.

b *Each value represents the average of triplicate measurements ± standard deviation. ND: not determined because failed in transfection study*.

c *Encapsulation efficiency*.

d *Preparation pH is 6.23*.

e *Preparation pH is 5.52*.

One noticeable observation in Table 4 is NP shrinkage upon loading siRNA, except in the case of CSP-LC-TPP NPs, which increased in size by *ca*. 40 nm upon loading (Tables 2, 4). The shrinkage was particularly obvious with CS-LC and CSP-LC NPs, which were size-reduced by an average of *ca*. 260 nm. On the other hand, CS-LC-TPP NPs sizes suffered only *ca*. 80 nm reduction upon loading of the siRNA cargo. Shrinkage suggests that loaded siRNA polyanion deposits at CS-LC and CSP-LC NP shells caused significant surface force onto NPs shells (*via* electrostatic attraction with cationic CS), thus promoting size reduction. This behavior has been previously reported (Liu et al., 2007). However, NP size reduction is less pronounced in CS-LC-TPP NPs, because TPP crosslinking promotes *a priori* significant NP size reduction, therefore, any additional NP shrinkage by loaded siRNA would become much less drastic (*ca*. 80 nm compared with *ca*. 260 nm upon loading CS-LC and CSP-LC NPs). On the other hand, the increase observed in CSP-LC-TPP NP sizes is suggestive of core loading (discussed below).

However, upon increasing the pH to 7.4, loaded CS-LC-TPP and CSP-LC-TPP NPs showed a significant increase in size (Table 4) albeit more pronounced in the case of CS-LC-TPP NPs (*ca*. 150 nm). This suggests that basic pH tends to deprotonate CS ammonium surface groups and weaken electrostatic attraction anchoring TPP to the NP surface with concomitant easing of tight surface packing and increase in NPs size. On the other hand, this effect is less drastic in CSP-based NPs, because the grafted phthalates provide a certain degree of surface crosslinking that compensates for TPP loss caused by pH change (Saeed et al., 2020).

Another interesting observation is related to the influence of siRNA loading on NP surface charges. All the NP formulas generally maintained significant positive surface charges upon loading at preparation pH (Tables 3, 4). However, upon increasing the pH to 7.4, both loaded and unloaded NPs suffered a significant loss in their positive surface charges. This effect was particularly evident in the case of CSP-LC-TPP NPs, which exhibited the most negative surface charge upon switching to pH 7.4. The reason for this trend is probably due to the deprotonation of CS and CSP ammonium moieties concomitant with basic conditions leaving TPP anions to dominate the NP surface. This effect is more pronounced in CSP-LC-TPP NPs because of negative charges accompanying the deprotonation of phthalate moieties on CSP-LC-TPP NP surfaces.

Interestingly, CSP-LC-TPP NPs maintained nearly constant surface charges upon siRNA loading, i.e., under comparable pH conditions, as in Tables 3, 4. For example, at pH 7.4 unloaded CSP-LC-TPP NPs exhibit a surface charge of −7.5 ± 0.4 mV to become −8.27 ± 0.91 mV upon loading (a similar trend is seen at preparation pH). On the other hand, siRNA loading significantly affected the surface charge of CS-LC-TPP NPs (e.g., at pH 7.4 the surface charge shifted from +4.3 ± 1.1 mV, as in Table 3, to −5.93 ± 1.02 mV, as in Table 4, a similar trend is also seen at preparation pH).

These trends reflect the notion suggested from the effect of siRNA loading on NP sizes: CS-LC-TPP NPs load their anionic siRNA cargo within the vicinity of their outer shells, causing the observed reduction in positive surface charge, while CSP-LC-TPP NPs load their cargo probably within their LC cores, explaining the lack of change in their surface charge upon loading. This conduct is in agreement with transfection results: CSP-LC-TPP NPs were superior to their CS-LC-TPP NP analogs (see **Figure 3**), because the latter has their cargo exposed to nucleases in the outer environment. We hypothesize that in CS-LC-TPP NPs, a significant fraction of added TPP complexes with LC cationic charge (choline fragments) to form NP cores (Pérez et al., 2012), while CS engulfs the complex, leaving significant NP positive surface charge available for complexation with siRNA cargo, which in turn reduce NP cationic surface charges upon loading. This proposition is in agreement with previous reports (Senel et al., 2015; Tezgel et al., 2018). On the other hand, in the case of CSP-LC-TPP NPs, LC forms a tighter complex with CSP mediated by electrostatic attraction with grafted anionic phthalate moieties, thus leaving TPP to reside significantly at NPs shells and rendering NP surfaces repulsive to siRNA. This leaves LC as the remaining viable interaction partner with anionic siRNA, thus pushing siRNA into NPs LC-rich cores. Still, any firm conclusions regarding core/shell loading warrant detailed future assessment.

### NPs Morphology

**Figure 2:**
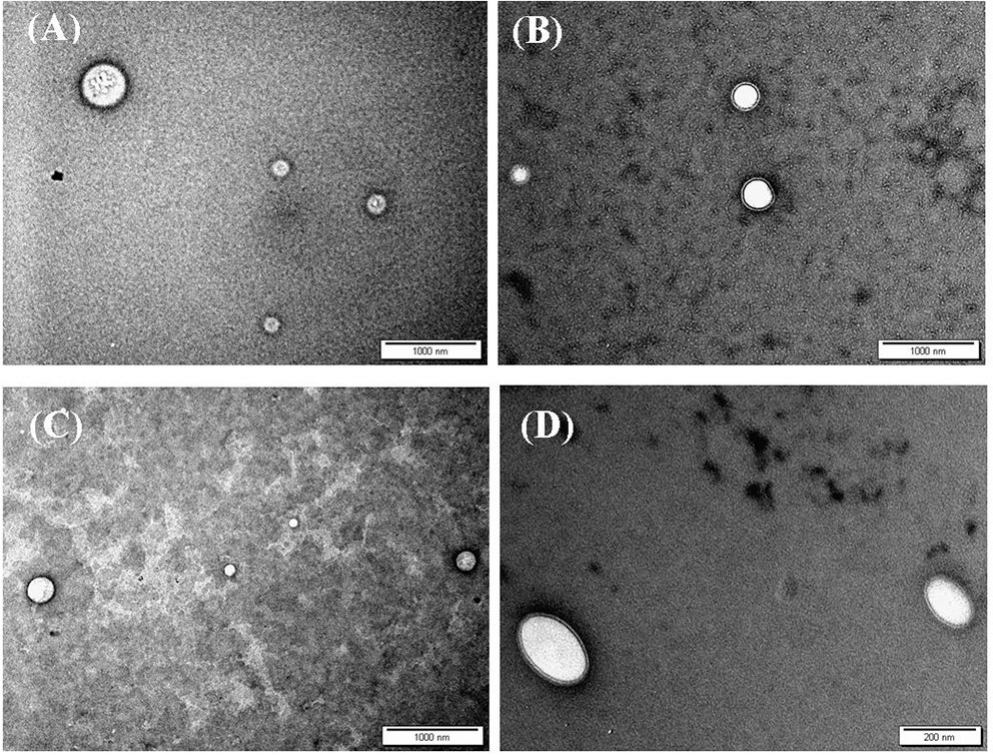
TEM images of siRNA loaded **(A)** CS-LC NPs, **(B)** CS-LC-TPP NPs, **(C)** CSP-LC NPs, and **(D)** CSP-LC-TPP NPs. More images are shown in Supplementary Figures 2, 3.

Transmission electron microscopy (TEM) was used to assess the morphological properties of siRNA-loaded NPs, as in Figure 2. Clearly, from the figure, NP sizes are within ranges identified by the DLS analysis (Table 4).

Interestingly, loaded TPP-based NPs, i.e., CS-LC-TPP and CSP-LC-TPP NPs, exhibit compact double-layer outer shell structures (Figures 2B,D) that are absent in the case of TPP-devoid NPs (CS-LC and CSP-LC NPs, Figures 2A,C), which exhibit diffuse shells. Apparently, TPP induces highly ordered double-layer shells composed of TPP-crosslinked CS (or CSP) with LC playing a certain role in their formation. This is the first time that such structure for CS-LC or any other CS-lipid nano-complexes has been reported. Another very interesting observation shown in Figure 2 is the ellipsoid shapes of CSP-LC-TPP NPs compared with the perfectly round spherical shapes of other NPs. This shape has not been reported, and we believe it is related to certain unique interactions involving TPP, conjugated phthalate, and LC. However, this observation warrants detailed future investigation. Needless to say, NP shape and orientation greatly influence cellular uptake (Dasgupta et al., 2014). It has been suggested that ellipsoid-shaped NPs have better chances of being taken up by cell membranes compared with their spherical counterparts (Deng et al., 2019), which, at least partially, explains the better cellular uptake and transfection efficiency of ellipsoid-shaped CSP-LC-TPP NPs.

### Transfection Studies

Sizes of loaded nanoparticles in physiologically relevant conditions, i.e., <350nm (Table 4) should allow them to selectively penetrate cancer tissues and to be up-taken passively by cancer cells (i.e., enhanced permeation and retention, EPR) without the need for any targeting motif(s) (Jain and Stylianopoulos, 2010; Maeda et al., 2013). In fact, EPR has become a mainstay of anticancer nano-drug delivery (Hashizume et al., 2000; Wilhelm et al., 2016). Accordingly, we evaluated the gene delivery efficiencies of loaded NPs *in vitro* by measuring the SLUG expression in MDA-MB-453 cancer cells after incubation with siRNA-loaded NPs. However, to closely simulate physiological conditions, we opted to carry out the transfection studies in the presence and absence of serum and at physiological pH. Serum is considered a significant challenge for siRNA transfection, because it includes nucleases that degrade unprotected RNAi (Choi et al., 2018).

**Figure 3:**
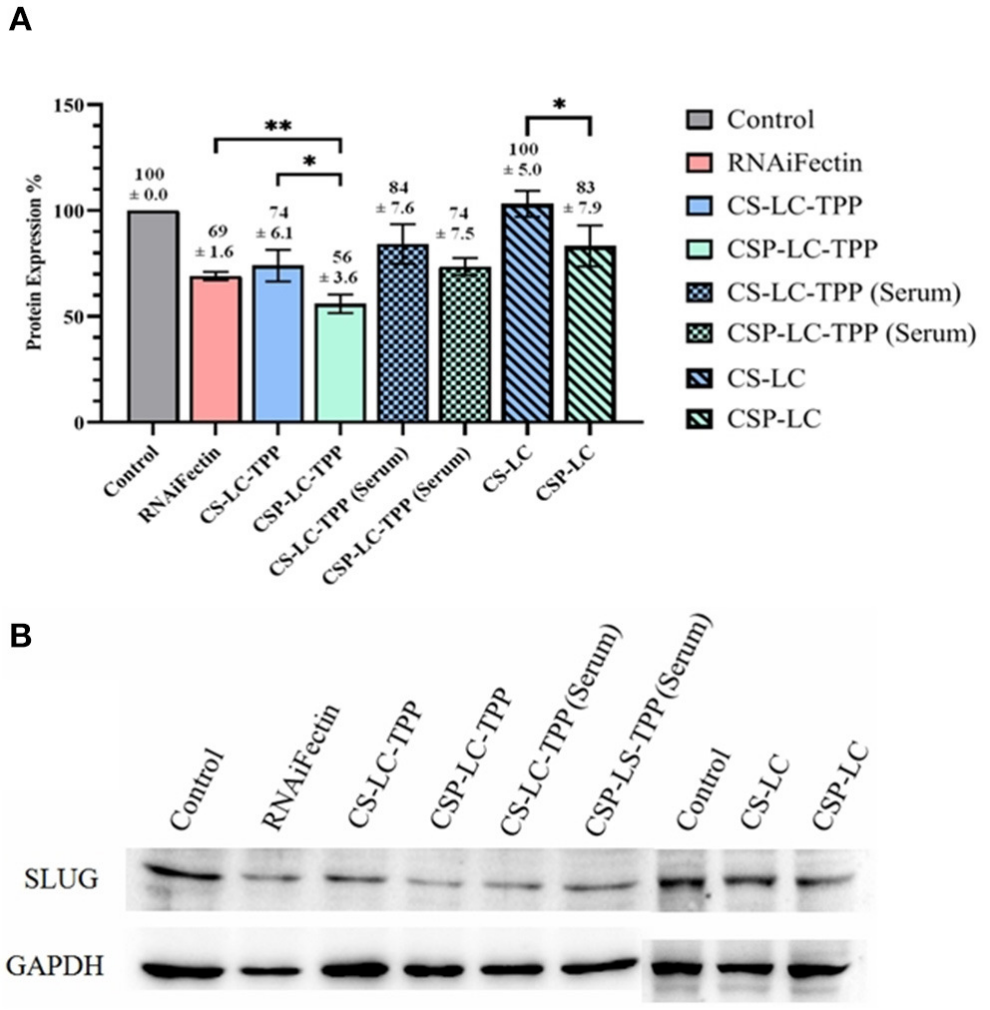
SLUG Protein silencing in response to anti-SLUG siRNA-loaded NPs and RNAiFectin. **(A)** Histogram showing SLUG protein expression in response to different NPs formulas. The numbers above each bar represent the average SLUG protein expression percentage (±SD) of triplicate trials. ns: no significant difference, *significant difference at *P* < 0.05, **significant difference at *P* < 0.01. **(B)** Image showing the gel electrophoresis membrane for Western blotting. GAPDH (glyceraldehyde-3-phosphate dehydrogenase) protein expression was used as loading control. Un-transfected cells were used as controls. Supplementary Figures 4A,B show the complete Western blot membranes.

Evidently from Figure 3, CSP-LC-TPP and CS-LC-TPP NPs scored the best silencing rates with corresponding SLUG expression rates reduced to 56 and 74%, respectively, under serum-deprived conditions. In the presence of serum, however, the same NP formulas scored 77 and 84% expression rates, respectively. Interestingly, under serum-free conditions, CSP-LC-TPP NPs scored better than the well-known commercial transfection carrier RNAifectin, which yielded a SLUG expression rate of 69%. RNAifectin is not effective at all in the presence of serum according to the manufacturer protocol.

As expected, the TPP-deprived NP formulas, i.e., CS-LC and CSP-LC, were much less effective compared with their TPP-crosslinked analogs. In fact, CS-LC NPs failed totally to reduce the expression of SLUG protein. CSP-LC scored better with a SLUG expression rate of 83% albeit in the absence of serum.

Clearly from Figure 3, the transfection efficiency of loaded-CSP-LC-TPP NPs superseded that of loaded-CS-LC-TPP NPs probably because of smaller size (*ca*. 254 nm at pH 7.4) and ellipsoid shape (Figure 2, Supplementary Figure 3) compared with loaded CS-LC-TPP NPs, which have a larger size (*ca*. 345 nm at pH 7.4) and spherical shape (Figure 2, Supplementary Figure 2).

Larger NPs are less-than-optimal nanocarriers for siRNA delivery, as they are less effectively up-taken by cells (Shao et al., 2015). Moreover, since CSP is amphoteric (because of its cationic chitosan amines and anionic phthalates), it should better promote the endosomal escape of siRNA compared with CS due to its anticipated superior buffering “proton sponge” capacity (Richard et al., 2013). This assumption is supported by reports indicating that amphoteric polymers have better transfection efficiency compared with their cationic analogs because of their enhanced pH buffering properties. It is hypothesized that polymeric buffers consume endosomal protons and, therefore, derive endosomal proton-chloride co-transporter to keep shuttling protons and chloride ions into endosomes leading to increased osmotic pressure, endosomal swelling, and eventually endosomal rupture and escape of siRNA cargo into the cellular cytoplasm (Oskuee et al., 2009; Richard et al., 2013; Ni et al., 2017; Ryu et al., 2018).

It is noteworthy to mention that loaded CSP-LC-TPP and CS-LC-TPP NPs exhibit comparable, slightly negative, surface charges under physiological pH conditions, suggesting that surface charges are not responsible for the observed transfection variations.

### Stability of siRNA-Loaded NPs

#### Stability in Serum

The fact that transfection data are based on *in vitro* experiments, it is important to assess the stability of NPs and their loaded siRNA under serum conditions for any future potential *in vivo* applications. Systemic (or serum) nucleases significantly decrease the plasma half-life of siRNA (Hannon and Rossi, 2004). Thus, it is critical for any nanocarrier system intended to deliver siRNA to provide long-term protection from nuclease degradation and allow to accumulate in targeted cells after systemic administration.

Gel electrophoresis was performed to assess the stability of loaded siRNA within CS-LC-TPP and CSP-LC-TPP NPs in the presence of 50% FBS at pH 6.8 and 7.4. These conditions should simulate the extracellular matrices of tumor and normal tissues (Wagner and Wiig, 2015), respectively. CS-LC-TPP and CSP-LC-TPP NPs were selected because they succeeded in transfection studies.

**Figure 4:**
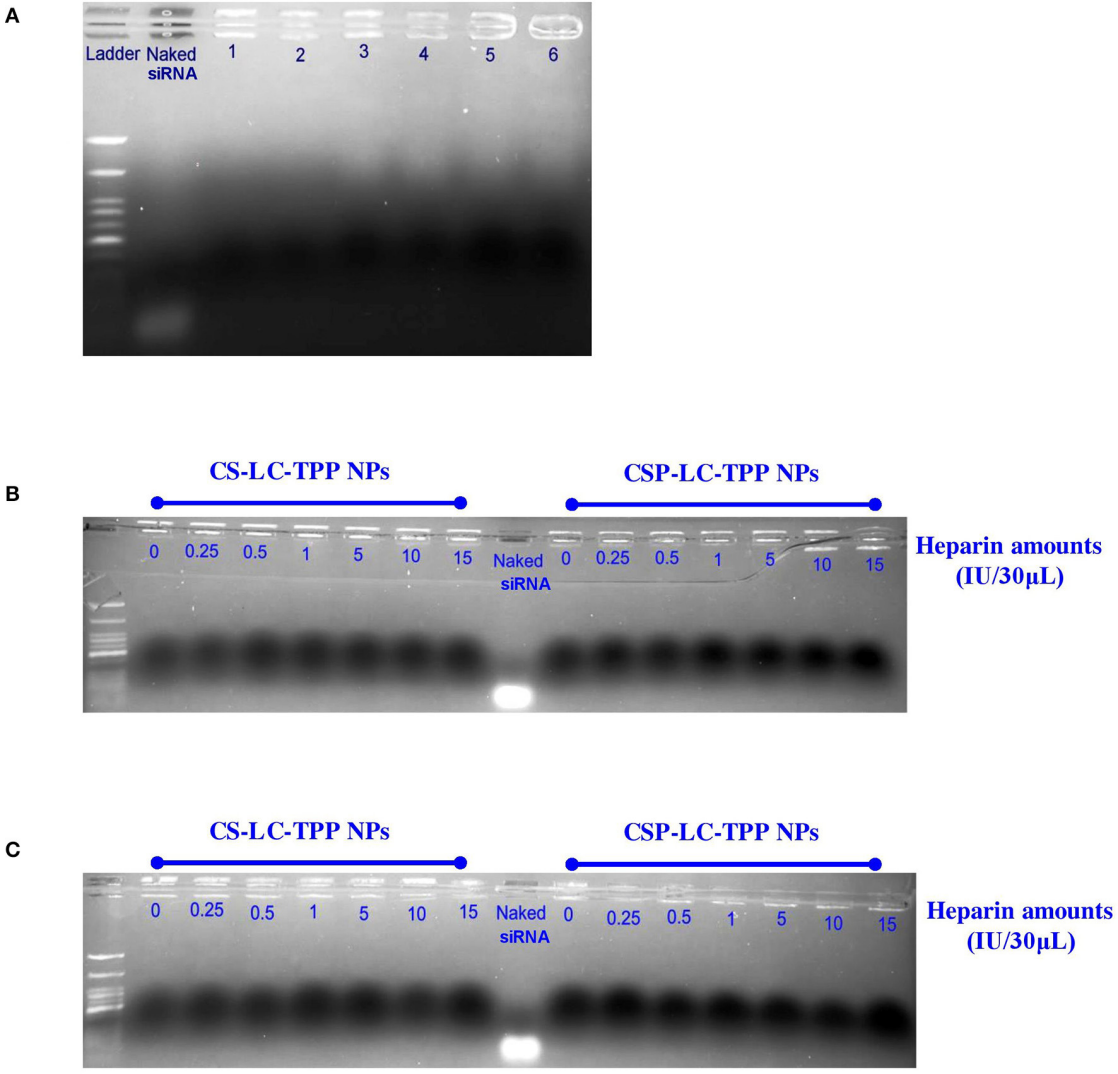
Gel electrophoresis bands for siRNA within loaded NPs: **(A)** CS-LC-TPP and CSP-LC-TPP NPs in aqueous solution at preparation pH (lanes 1 and 2, respectively), at pH 6.8 in 50% FBS solution (lanes 3 and 4, respectively) and at pH 7.4 in 50% FBS solution (lanes 5 and 6, respectively), **(B,C)** siRNA-loaded CS-LC-TPP and CSP-LC-TPP NPs in different heparin concentrations at pH 6.8 and 7.4 PBS, respectively. Naked siRNA was used as positive control.

Figure 4A shows the results of the experiment. Clearly from the figure, in contrast to the naked siRNA band, which migrated significantly on the agarose gel, the electrophoresis bands corresponding to loaded siRNA remained at the gel baseline regardless of whether the corresponding NPs were exposed to acidic (6.8 and preparation pH, Table 4), neutral (pH 7.4), or FBS conditions. Still, baseline band intensities were slightly lighter for FBS-exposed NPs compared with their FBS-unexposed counterparts, suggesting only slight FBS-mediated RNA degradation. This agrees with the lesser transfection efficiencies observed for CS-LC-TPP and CSP-LC-TPP NPs in the presence of FBS compared with FBS-lacking conditions, as in Figure 3. This behavior highlights the capacity of the NPs to protect loaded siRNA for at least 2 h (time of exposure to different pH or FBS conditions), suggesting tight and protective NP surfaces.

#### Stability to Heparin-siRNA Displacement

Polyanions were reported to disassemble DNA or siRNA polyplexes *in vitro* (Bertschinger et al., 2006). Thus, heparin sulfate, which is a polyanion normally attached to cell surface proteoglycans, has the potential to displace loaded RNA and disassemble corresponding loaded NPs, leading to premature release of siRNA upon interactions with cell surfaces. Accordingly, we decided to study the RNA protective capabilities of CS-LC-TPP and CSP-LC-TPP NPs in the presence of heparin.

The study commenced by exposing siRNA-loaded CS-LC-TPP and CSP-LC-TPP NPs to variable heparin concentrations (0–15 IU/30 μl) at two pH conditions (6.8 and 7.4) and 37°C for over 3 h. Thereafter, the tested NPs were applied onto the gel and analyzed by gel electrophoresis.

Figures 4B,C show the results. Clearly, the electrophoretic bands of loaded RNA remained at the baseline despite the exposure of the loading NPs to different heparin concentrations and pH conditions. In contrast, naked siRNA exhibited significant electrophoretic migration.

These results further support the notion about the surface tightness and cohesiveness of the loaded CS-LC-TPP and CSP-LC-TPP NPs. Moreover, these findings are in agreement with the reported ability of chitosan-based NPs to protect siRNA from being displaced by heparin (Rastegari et al., 2019).

### NPs Cellular Uptake and Intracellular Release of siRNA

Although the prepared nanoparticles successfully transfected the targeted cells, transfection itself requires no more than 1–2% of loaded siRNA to enter the RNAi machinery (Gilleron et al., 2013; Sahay et al., 2013). Therefore, it can be argued that transfection does not guarantee the integrity of the nanocarrier system within the cellular cytoplasm. Accordingly, we were interested to probe the intercellular integrity of the nanocarrier system. We opted for the fluorescent siRNA (siRNA AF488) cargo as well as a second extremely water-soluble drug cargo, i.e., DOX. It should be very hard to contain DOX in less-than-optimal or partially degraded intracellular nanoparticles. Additionally, DOX is fluorescent and should, therefore, be easily monitored intracellularly (Saeed et al., 2020). Thereafter, confocal fluorescence microscopy was performed to study the uptake of NPs loaded with either cargo (DOX or siRNA) by MDA-MB-453 cancer cells. We opted to evaluate only CS-LC-TPP and CSP-LC-TPP NPs because they retained satisfactory stability at physiological pH (Table 3) and exhibited the best transfection efficiencies compared with the other NP formulas (Figure 3). Free DOX, naked fluorescent siRNA, RNAifectin loaded with fluorescent siRNA, and untreated cells were used as controls.

**Figure 5:**
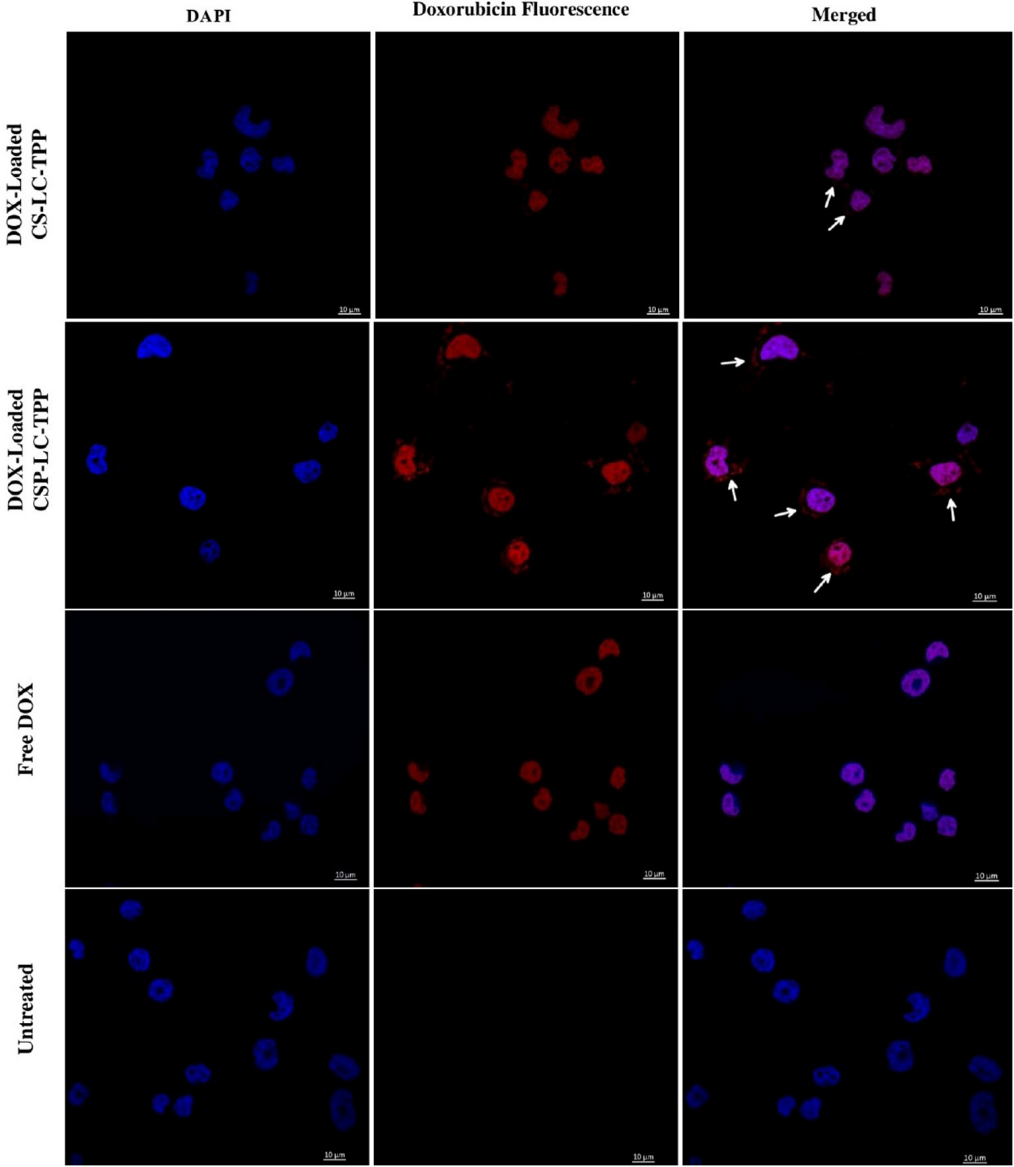
Confocal fluorescence microscopy images showing MDA-MB-453 cancer cells treated with DOX-loaded CS-LC-TPP NPs, CSP-LC-TPP NPs, free DOX, and control (untreated) cells. All treatments are equivalent to 1 μM doxorubicin over 4-h periods. White arrows point nanoparticles uptaken into cellular cytoplasm. Scale = 10 μm.

**Figure 6:**
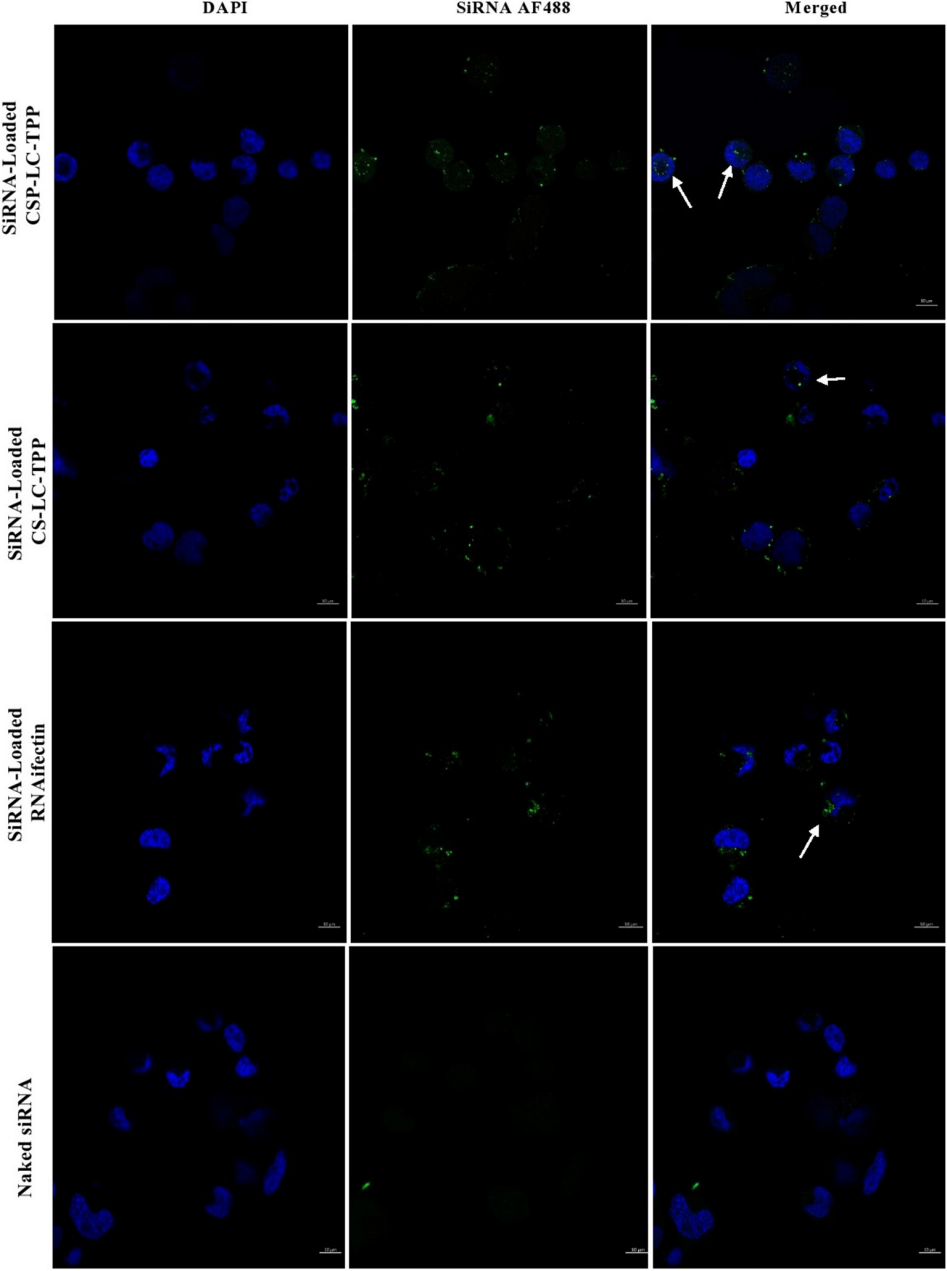
Confocal fluorescence microscopy images showing MDA-MB-453 cancer cells treated with fluorescent siRNA AF488-loaded CS-LC-TPP NPs and CSP-LC-TPP NPs. RNAifectin loaded with fluorescent siRNA AF488 and naked siRNA AF488 were studied as controls. All treatments are equivalent to 50 nM siRNA over 4-h periods. White arrows point nanoparticles up taken into cellular cytoplasm. Scale = 10 μm.

Figures 5, 6 show the results of the confocal microscopy studies. It should be mentioned that spot-like fluorescent structures observed in cellular cytoplasms indicate intact loaded nanoparticles, while diffuse fluorescence arises from the endosomal release of cargo (Cardarelli et al., 2016).

Figure 5 shows that contrary to untreated cells, drug-loaded NPs and free DOX caused cellular nuclei to fluoresce, indicating nuclear uptake of released free DOX. Figures 5, 6 clearly demonstrate significant internalization of DOX- and siRNA-loaded CSP-LC-TPP NPs compared with their CS-LC-TPP counterparts, which exhibited less evident uptake. These results are rather anticipated and agree with the transfection results shown in Figure 3. The fact that both NP formulas have comparable stability under variable pH conditions (Table 2) suggests that the better uptake of CSP-LC-TPP NPs was probably due to their ellipsoid shapes (Figure 2D) and smaller sizes (*ca*. 186 nm) at physiological pH compared with the spherical shapes (Figure 2C) and larger sizes (*ca*. 239.5 nm) of CS-LC-TPP NPs at same pH (Tables 2, 3). Additionally, Figure 6 also shows siRNA-loaded RNAifectin to exhibit comparable cellular uptake compared with CSP-LC-TPP counterparts. Unsurprisingly, naked siRNA failed to penetrate cancer cells, as in Figure 6, probably because of its hydrophilic nature and chemical instability (He et al., 2019).

**Figure 7:**
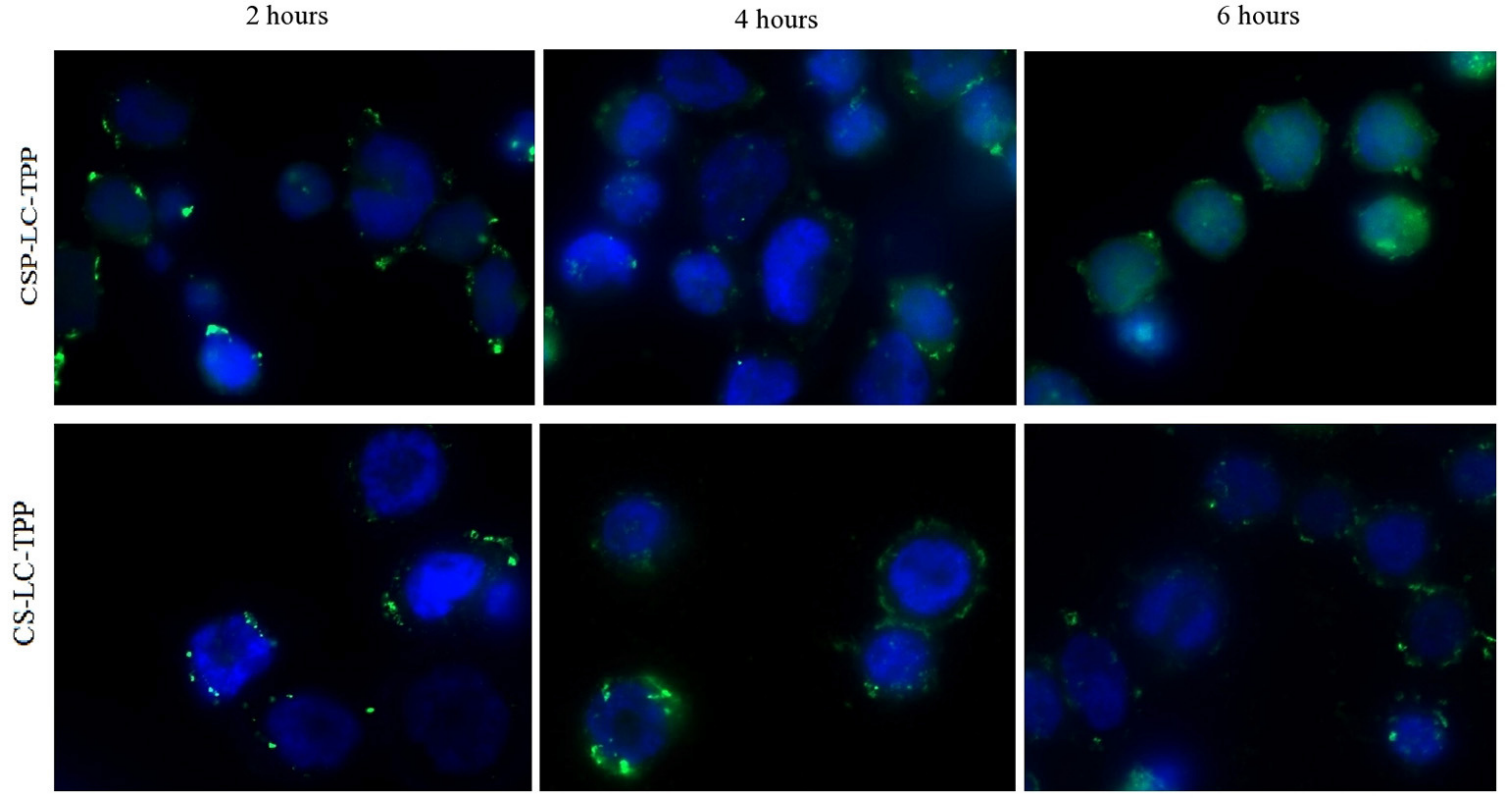
Wide field fluorescence microscopy images showing MDA-MB-453 cancer cells treated with fluorescent siRNA (AF488)-loaded CS-LC-TPP NPs and CSP-LC-TPP NPs (green) merged with DAPI fluorescence for cells nucleus (blue). All treatments are equivalent to 50 nM siRNA over 2-, 4-, and 6-h periods.

Figure 7 shows the cellular uptake and intracellular release of siRNA from CS-LC-TPP and CSP-LC-TPP NPs as a function of time. Three-time steps were sampled, namely, at 2, 4, and 6 h. Clearly from Figure 7, NPs from both formulations were successfully uptaken after 2 h, as evidenced by the intense intracellular fluorescent dots. After 4 h, however, the intracellular fluorescent dots remained intact albeit of lesser intensity, suggesting the partial release of the siRNA cargo. Interestingly, after 6 h, the image corresponding to CSP-LC-TPP NPs shows significant intracellular diffusion of fluorescence contrary to CS-LC-TPP NPs which exhibit more robust intracellular florescent agglomerates. This indicates that CSP-LC-TPP NPs successfully released their siRNA content after 6 h, while their CS-LC-TPP NP counterparts were less successful in doing so. Such findings are in agreement with the formal explanation about the superior transfection capacity of CSP-LC-TPP NPs (section Transfection Studies): the superior ability of amphoteric CSP to act as a buffering “proton sponge” (i.e., compared with the largely basic CS) causes eventual endosomal rupture and escape of the siRNA cargo into the cellular cytoplasm.

### Cytotoxicity Study

**Figure 8:**
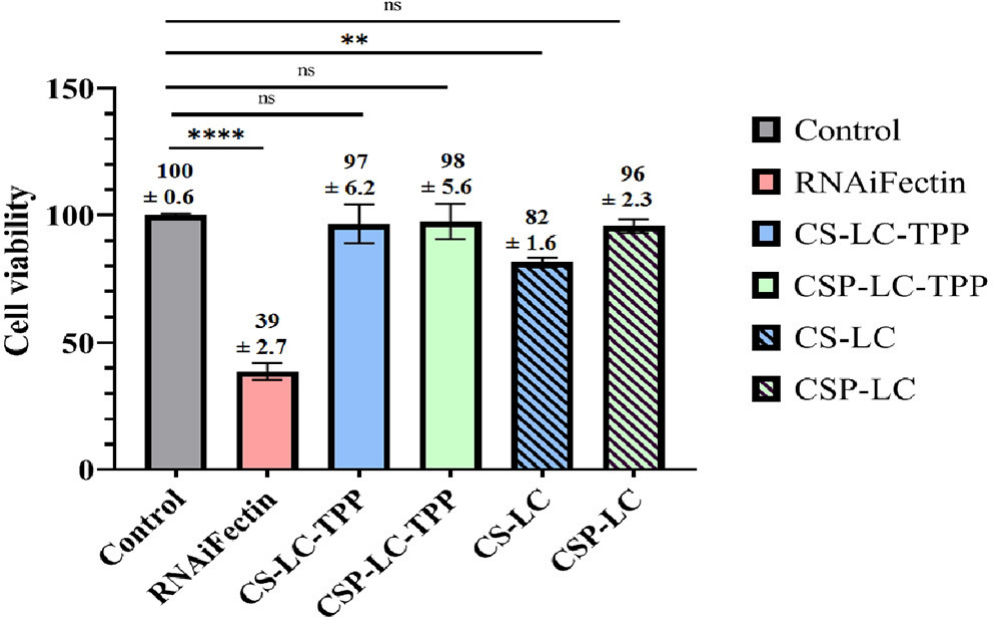
Histogram showing the percentage of MDA-MB-453 cancer cell viability upon exposure to NPs and RNAifectin. ns: no significant difference, **significant difference at *P* < 0.005, ****significant difference at *P* < 0.0001. Untreated cells used as control.

It is important to investigate if NPs would cause any cytotoxicity as they deliver their cargo intracellularly. Hence, MDA-MB-453 cells were exposed to the different unloaded NP formulas as well as a commercial liposomal transfection reagent (RNAifectin), and then the cell viability was assessed (Figure 8). Clearly, CSP-LC, CS-LC-TPP, and CSP-LC-TPP NPs had negligible cell cytotoxicities with cell viabilities of 96 97, and 98%, respectively, but CS-LC NPs were more cytotoxic with cell viability of 82%. On the other hand, the commercial carrier, RNAifectin, was found to be significantly cytotoxic with cell viability of only 39%. Apparently, the cytotoxicity of CS-LC NPs is related to their stronger cationic surface charge compared with the other NP formulas that exhibit slightly positive or even negative surface charges (i.e., CSP-LC-TPP NPs, Table 3). Strongly cationic NPs are reported to be significantly cytotoxic compared with anionic or slightly cationic NPs, since they tend to complex and disrupt the cellular anionic phospholipid bilayer membrane (Shao et al., 2015). Importantly, the most efficient transfection carrier CSP-LC-TPP NPs are virtually non-cytotoxic and, therefore, very appropriate for siRNA delivery. Supplementary Figure 5 shows light microscopy images of cellular cytotoxicity.

## Conclusion

A new synthetic siRNA delivery vector has been introduced herein based on CSP-LC-TPP NPs. The new NPs showed excellent stability under physiologically relevant conditions (pH 7.4 and 10% FBS), with a size range of *ca*. 185 nm and a surface charge of −7.5 mV at physiological pH. The TEM analysis indicates that the new NPs have peculiar ellipsoid morphology. Cellular uptake and transfection studies highlight the superiority of the new NP formula over other related NP compositions. They are even superior to the commercial transfection agent—RNAifectin—in silencing SLUG protein synthesis in cancer cells. Additionally, CSP-LC-TPP NPs were found to be virtually non-cytotoxic. However, despite success in delivering siRNA into cancer cells, CSP-TPP-LC NPs need to be fully investigated *in vivo* to be successfully implemented within clinical settings.

## Data Availability Statement

The original contributions presented in the study are included in the article/Supplementary Material, further inquiries can be directed to the corresponding author/s.

## Author Contributions

MT and MAh: supervision and administration. RS and MT: study design, conceptualization, and analysis and interpretation of data. RS and MAb: experimental work, methodology, software, and data curation. MT, MAh, RS, and MAb: visualization, investigation, and results validation: RS: writing of the original manuscript. MT, RS, and MAh: editing of the manuscript. All the authors reviewed and approved the final version of the manuscript.

## Conflict of Interest

The authors declare that the research was conducted in the absence of any commercial or financial relationships that could be construed as a potential conflict of interest.

## Publisher's Note

All claims expressed in this article are solely those of the authors and do not necessarily represent those of their affiliated organizations, or those of the publisher, the editors and the reviewers. Any product that may be evaluated in this article, or claim that may be made by its manufacturer, is not guaranteed or endorsed by the publisher.

## Abbreviations

NPs: nanoparticles
CS: chitosan
CSP: chitosan phthalate
LC: lecithin
TPP: tripolyphosphate
CSP-LC-TPP: chitosan phthalate lecithin tripolyphosphate
CS-LC-TPP: chitosan lecithin tripolyphosphate
siRNA: short interfering ribonucleic acid
SiRNA AF488: short interfering ribonucleic acid with Alexa Fluor 488
DOX: doxorubicin
FBS: fetal bovine serum
DLS: dynamic light scattering
PDI: polydispersity index
TEM: transmission electron microscopy.

## References

[B1] Abozeid S. M. Hathout R. M. Abou-Aisha K. (2016). Silencing of the metastasis-linked gene, AEG-1, using siRNA-loaded cholamine surface-modified gelatin nanoparticles in the breast carcinoma cell line MCF-7. Colloids Surf. B Biointerfaces 145, 607–616. 10.1016/j.colsurfb.2016.05.066 27285732

[B2] Abulateefeh S. R. Taha M. O. (2015). Enhanced drug encapsulation and extended release profiles of calcium–alginate nanoparticles by using tannic acid as a bridging cross-linking agent. J. Microencapsul. 32, 96–105. 10.3109/02652048.2014.985343 25413187

[B3] Ahram M. Mustafa E. Abu Hammad S. Alhudhud M. Bawadi R. Tahtamouni L.. (2018). The cellular and molecular effects of the androgen receptor agonist, Cl-4AS-1, on breast cancer cells. Endocr. Res. 43, 203–214. 10.1080/07435800.2018.1455105 29578828

[B4] Alameh M. Lavertu M. Tran-Khanh N. Chang C.-Y. Lesage F. Bail M.. (2018). siRNA delivery with chitosan: influence of chitosan molecular weight, degree of deacetylation, and amine to phosphate ratio on *in vitro* silencing efficiency, hemocompatibility, biodistribution, and *in vivo* efficacy. Biomacromolecules 19, 112–131. 10.1021/acs.biomac.7b01297 29211954

[B5] Baghdan E. Pinnapireddy S. R. Strehlow B. Engelhardt K. H. Schäfer J. Bakowsky U. (2018). Lipid coated chitosan-DNA nanoparticles for enhanced gene delivery. Int. J. Pharm. 535, 473–479. 10.1016/j.ijpharm.2017.11.045 29175439

[B6] Bertschinger M. Backliwal G. Schertenleib A. Jordan M. Hacker D. L. Wurm F. M. (2006). Disassembly of polyethylenimine-DNA particles *in vitro*: implications for polyethylenimine-mediated DNA delivery. J. Control. Release 116, 96–104. 10.1016/j.jconrel.2006.09.006 17079047

[B7] Besin G. Milton J. Sabnis S. Howell R. Mihai C. Burke K.. (2019). Accelerated blood clearance of lipid nanoparticles entails a biphasic humoral response of B-1 followed by B-2 lymphocytes to distinct antigenic moieties. ImmunoHorizons 3, 282–293. 10.4049/immunohorizons.1900029 31356158

[B8] Böker K. Schneider S. Gruber J. Komrakova M. Sehmisch S. Lehmann W.. (2019). New gene therapy approach: prelonged virus-like particle mediated RANKL knockdown using chitosan hydrogels. Osteologie 28, V 8.V 2. 10.1055/s-0039-1680011

[B9] Bono N. Ponti F. Mantovani D. Candiani G. (2020). Non-viral *in vitro* gene delivery: it is now time to set the bar! *Pharmaceutics* 12:183. 10.3390/pharmaceutics12020183 PMC7076396 32098191

[B10] Cao Y. Tan Y. F. Wong Y. S. Liew M. W. J. Venkatraman S. (2019). Recent advances in chitosan-based carriers for gene delivery. Mar. Drugs 17:381. 10.3390/md17060381 31242678 PMC6627531

[B11] Cao Y. W. Wan G. X. Sun J. P. Cui X. B. Hu J. M. Liang W. H.. (2015). Implications of the Notch1-Snail/Slug-epithelial to mesenchymal transition axis for lymph node metastasis in infiltrating ductal carcinoma. Kaohsiung J. Med. Sci. 31, 70–76. 10.1016/j.kjms.2014.11.008 25645984 PMC11916146

[B12] Cardarelli F. Digiacomo L. Marchini C. Amici A. Salomone F. Fiume G.. (2016). The intracellular trafficking mechanism of Lipofectamine-based transfection reagents and its implication for gene delivery. Sci. Rep. 6:25879. 10.1038/srep25879 27165510 PMC4863168

[B13] Carpenter R. L. Paw I. Dewhirst M. W. Lo H.-W. (2015). Akt phosphorylates and activates HSF-1 independent of heat shock, leading to Slug overexpression and epithelial–mesenchymal transition (EMT) of HER2-overexpressing breast cancer cells. Oncogene 34, 546–557. 10.1038/onc.2013.582 24469056 PMC4112182

[B14] Chadha R. Gupta S. Pathak N. (2012). Artesunate-loaded chitosan/lecithin nanoparticles: preparation, characterization, and *in vivo* studies. Drug Dev. Ind. Pharm. 38, 1538–1546. 10.3109/03639045.2012.658812 22348223

[B15] Chen C.-K. Huang P.-K. Law W.-C. Chu C.-H. Chen N.-T. Lo L.-W. (2020). Biodegradable polymers for gene-delivery applications. Int. J. Nanomed. 15:2131. 10.2147/IJN.S222419 PMC7125329 32280211

[B16] Choi C. K. K. Zhang L. Choi C. H. J. (2018). Efficient siRNA delivery with non-cationic carriers. Nat. Biomed. Eng. 2, 275–276. 10.1038/s41551-018-0240-z 30936456

[B17] Chu X. Y. Huang W. Wang Y. L. Meng L. W. Chen L. Q. Jin M. J.. (2019). Improving antitumor outcomes for palliative intratumoral injection therapy through lecithin– chitosan nanoparticles loading paclitaxel– cholesterol complex. Int. J. Nanomed. 14, 689–705. 10.2147/IJN.S188667 30774330 PMC6361321

[B18] Correa V. L. R. Martins J. A. de Souza T. R. Rincon G. de C. N. Miguel M. P.. (2020). Melatonin loaded lecithin-chitosan nanoparticles improved the wound healing in diabetic rats. Int. J. Biol. Macromol. 162, 1465–1475. 10.1016/j.ijbiomac.2020.08.027 32781118

[B19] Danhier F. Messaoudi K. Lemaire L. Benoit J.-P. Lagarce F. (2015). Combined anti-Galectin-1 and anti-EGFR siRNA-loaded chitosan-lipid nanocapsules decrease temozolomide resistance in glioblastoma: *in vivo* evaluation. Int. J. Pharm. 481, 154–161. 10.1016/j.ijpharm.2015.01.051 25644286

[B20] Dasgupta S. Auth T. Gompper G. (2014). Shape and orientation matter for the cellular uptake of nonspherical particles. Nano Lett. 14, 687–693. 10.1021/nl403949h 24383757

[B21] De Jesus M. B. Zuhorn I. S. (2015). Solid lipid nanoparticles as nucleic acid delivery system: Properties and molecular mechanisms. J. Control. Release 201, 1–13. 10.1016/j.jconrel.2015.01.010 25578828

[B22] Delgado D. del Pozo-Rodríguez A. Angeles Solinís M. Bartkowiak A. Rodríguez-Gascón A. (2013). New gene delivery system based on oligochitosan and solid lipid nanoparticles: ‘*in vitro*' and ‘*in vivo*' evaluation. Eur. J. Pharm. Sci. 50, 484–491. 10.1016/j.ejps.2013.08.013 23981333

[B23] Deng H. Dutta P. Liu J. (2019). Entry modes of ellipsoidal nanoparticles on a membrane during clathrin-mediated endocytosis. Soft Matter 15, 5128–5137. 10.1039/C9SM00751B 31190048 PMC7570437

[B24] Dmour I. Taha M. O. (2017). Novel nanoparticles based on chitosan-dicarboxylate conjugates via tandem ionotropic/covalent crosslinking with tripolyphosphate and subsequent evaluation as drug delivery vehicles. Int. J. Pharm. 529, 15–31. 10.1016/j.ijpharm.2017.06.061 28634140

[B25] Farid M. M. Hathout R. M. Fawzy M. Abou-Aisha K. (2014). Silencing of the scavenger receptor (Class B - Type 1) gene using siRNA-loaded chitosan nanaoparticles in a HepG2 cell model. Colloids Surf. B. Biointerfaces 123, 930–937. 10.1016/j.colsurfb.2014.10.045 25466457

[B26] Fiume M. Z. (2001). Final report on the safety assessment of Lecithin and Hydrogenated Lecithin. Int. J. Toxicol. 20, 21–45. 10.1080/109158101750300937 11358109

[B27] Fürst W. Banerjee A. (2005). Release of glutaraldehyde from an albumin-glutaraldehyde tissue adhesive causes significant *in vitro* and *in vivo* toxicity. Ann. Thorac. Surg. 79, 1522–1528. 10.1016/j.athoracsur.2004.11.054 15854927

[B28] Ganesan R. Mallets E. Gomez-Cambronero J. (2016). The transcription factors Slug (SNAI2) and Snail (SNAI1) regulate phospholipase D (PLD) promoter in opposite ways towards cancer cell invasion. Mol. Oncol. 10, 663–676. 10.1016/j.molonc.2015.12.006 26781944 PMC4870114

[B29] Gilleron J. Querbes W. Zeigerer A. Borodovsky A. Marsico G. Schubert U.. (2013). Image-based analysis of lipid nanoparticle-mediated siRNA delivery, intracellular trafficking and endosomal escape. Nat. Biotechnol. 31, 638–646. 10.1038/nbt.2612 23792630

[B30] Hannon G. J. Rossi J. J. (2004). Unlocking the potential of the human genome with RNA interference. Nature 431, 371–378. 10.1038/nature02870 15372045

[B31] Hashizume H. Baluk P. Morikawa S. McLean J. W. Thurston G. Roberge S.. (2000). Openings between defective endothelial cells explain tumor vessel leakiness. Am. J. Pathol. 156, 1363–1380. 10.1016/S0002-9440(10)65006-7 10751361 PMC1876882

[B32] He X. Yin F. Wang D. Xiong L.-H. Kwok R. T. K. Gao P. F.. (2019). AIE featured inorganic–organic core@ shell nanoparticles for high-efficiency siRNA delivery and real-time monitoring. Nano Lett. 19, 2272–2279. 10.1021/acs.nanolett.8b04677 30829039

[B33] Hu B. Zhong L. Weng Y. Peng L. Huang Y. Zhao Y.. (2020). Therapeutic siRNA: state of the art. Signal Transduct. Target. Ther. 5:101. 10.1038/s41392-020-0207-x 32561705 PMC7305320

[B34] Islam N. Dmour I. Taha M. O. (2019). Degradability of chitosan micro/nanoparticles for pulmonary drug delivery. Heliyon 5:e01684. 10.1016/j.heliyon.2019.e01684 31193324 PMC6525292

[B35] Jain R. K. Stylianopoulos T. (2010). Delivering nanomedicine to solid tumors. Nat. Rev. Clin. Oncol. 7, 653–664. 10.1038/nrclinonc.2010.139 20838415 PMC3065247

[B36] Jardim K. V. Siqueira J. L. N. Báo S. N. Sousa M. H. Parize A. L. (2020). The role of the lecithin addition in the properties and cytotoxic activity of chitosan and chondroitin sulfate nanoparticles containing curcumin. Carbohydr. Polym. 227:115351. 10.1016/j.carbpol.2019.115351 31590861

[B37] Kargaard A. Sluijter J. P. G. Klumperman B. (2019). Polymeric siRNA gene delivery – transfection efficiency versus cytotoxicity. J. Control. Release 316, 263–291. 10.1016/j.jconrel.2019.10.046 31689462

[B38] Ki M.-H. Kim J.-E. Lee Y.-N. Noh S. M. An S.-W. Cho H.-J.. (2014). Chitosan-based hybrid nanocomplex for siRNA delivery and its application for cancer therapy. Pharm. Res. 31, 3323–3334. 10.1007/s11095-014-1422-3 24858398

[B39] Kim B. Park J. H. Sailor M. J. (2019). Rekindling RNAi therapy: materials design requirements for *in vivo* siRNA delivery. Adv. Mater. 31, 1–23. 10.1002/adma.201903637 31566258 PMC6891135

[B40] Li T. Huang L. Yang M. (2020). Lipid-based vehicles for siRNA delivery in biomedical field. Curr. Pharm. Biotechnol. 21, 3–22. 10.2174/1389201020666190924164152 31549951

[B41] Liu X. Howard K. A. Dong M. Andersen M. Ø. Rahbek U. L. Johnsen M. G.. (2007). The influence of polymeric properties on chitosan/siRNA nanoparticle formulation and gene silencing. Biomaterials 28, 1280–1288. 10.1016/j.biomaterials.2006.11.004 17126901

[B42] Lostalé-Seijo I. Montenegro J. (2018). Synthetic materials at the forefront of gene delivery. Nat. Rev. Chem. 2, 258–277. 10.1038/s41570-018-0039-1

[B43] Maeda H. Nakamura H. Fang J. (2013). The EPR effect for macromolecular drug delivery to solid tumors: Improvement of tumor uptake, lowering of systemic toxicity, and distinct tumor imaging *in vivo*. Adv. Drug Deliv. Rev. 65, 71–79. 10.1016/j.addr.2012.10.002 23088862

[B44] Malfanti A. Scomparin A. Pozzi S. Gibori H. Krivitsky A. Blau R.. (2019). Oligo-guanidyl targeted bioconjugates forming rod shaped polyplexes as a new nanoplatform for oligonucleotide delivery. J. Control. Release 310, 58–73. 10.1016/j.jconrel.2019.08.005 31400381

[B45] Mansouri S. Lavigne P. Corsi K. Benderdour M. Beaumont E. Fernandes J. C. (2004). Chitosan-DNA nanoparticles as non-viral vectors in gene therapy: strategies to improve transfection efficacy. Eur. J. Pharm. Biopharm. 57, 1–8. 10.1016/S0939-6411(03)00155-3 14729076

[B46] Martin T. A. Goyal A. Watkins G. Jiang W. G. (2005). Expression of the transcription factors snail, slug, and twist and their clinical significance in human breast cancer. Ann. Surg. Oncol. 12, 488–496. 10.1245/ASO.2005.04.010 15864483

[B47] Murthy A. Ravi P. R. Kathuria H. Vats R. (2020). Self-assembled lecithin-chitosan nanoparticle improve the oral bioavailability and alter the pharmacokinetics of raloxifene. Int. J. Pharm. 588:119731. 10.1016/j.ijpharm.2020.119731 32763388

[B48] Nguyen J. Szoka F. C. (2012). Nucleic acid delivery: the missing pieces of the puzzle? Acc. Chem. Res. 45, 1153–1162. 10.1021/ar3000162 22428908 PMC3399092

[B49] Ni S. Xie Y. Tang Y. Liu Y. Chen J. Zhu S. (2017). Nebulized anionic guanidinylated O-carboxymethyl chitosan/N-2-hydroxypropyltimehyl ammonium chloride chitosan nanoparticles for siRNA pulmonary delivery: preparation, characterization and *in vitro* evaluation. J. Drug Target. 25, 451–462. 10.1080/1061186X.2016.1278219 28110554

[B50] Oskuee R. K. Dehshahri A. Shier W. T. Ramezani M. (2009). Alkylcarboxylate grafting to polyethylenimine: a simple approach to producing a DNA nanocarrier with low toxicity. J. Gene Med. 11, 921–932. 10.1002/jgm.1374 19634133

[B51] Pellá M. C. G. De Lima H. H. C. Rinaldi A. W. Fajardo A. R. Tenório-Neto E. T. Guilherme M. R.. (2020). Chitosan-based hydrogels for drug delivery. 163–190, 10.1007/978-981-15-0263-7_6

[B52] Pérez S. E. Gándola Y. Carlucci A. M. González L. Turyn D. Bregni C. (2012). Formulation strategies, characterization, and *in vitro* evaluation of lecithin-based nanoparticles for siRNA delivery. J. Drug Deliv. 2012, 1–9. 10.1155/2012/986265 22570790 PMC3335242

[B53] Pinnapireddy S. R. Raafat El Assy M. Schlote P. Bakowsky U. (2019). Glycosylated artificial virus-like hybrid vectors for advanced gene delivery. Polymers 11:243. 10.3390/polym11020243 30960227 PMC6419053

[B54] Ragelle H. Riva R. Vandermeulen G. Naeye B. Pourcelle V. Le Duff C. S.. (2014). Chitosan nanoparticles for siRNA delivery: optimizing formulation to increase stability and efficiency. J. Control. Release 176, 54–63. 10.1016/j.jconrel.2013.12.026 24389132

[B55] Rai R. Alwani S. Badea I. (2019). Polymeric nanoparticles in gene therapy: new avenues of design and optimization for delivery applications. Polymers 11:745. 10.3390/polym11040745 31027272 PMC6523186

[B56] Ramasamy T. Tran T. H. Choi J. Y. Cho H. J. Kim J. H. Yong C. S.. (2014). Layer-by-layer coated lipid–polymer hybrid nanoparticles designed for use in anticancer drug delivery. Carbohydr. Polym. 102, 653–661. 10.1016/j.carbpol.2013.11.009 24507332

[B57] Rao N. M. (2010). Cationic lipid-mediated nucleic acid delivery: beyond being cationic. Chem. Phys. Lipids 163, 245–252. 10.1016/j.chemphyslip.2010.01.001 20060819

[B58] Rastegari A. Mottaghitalab F. Dinarvand R. Amini M. Arefian E. Gholami M.. (2019). Inhibiting hepatic gluconeogenesis by chitosan lactate nanoparticles containing CRTC2 siRNA targeted by poly (ethylene glycol)-glycyrrhetinic acid. Drug Deliv. Transl. Res. 9, 694–706. 10.1007/s13346-019-00618-1 30825078

[B59] Richard I. Thibault M. De Crescenzo G. Buschmann M. D. Lavertu M. (2013). Ionization behavior of chitosan and chitosan-DNA polyplexes indicate that chitosan has a similar capability to induce a proton-sponge effect as PEI. Biomacromolecules 14, 1732–1740. 10.1021/bm4000713 23675916

[B60] Ridolfi D. M. Marcato P. D. Justo G. Z. Cordi L. Machado D. Durán N. (2012). Chitosan-solid lipid nanoparticles as carriers for topical delivery of tretinoin. Colloids Surf. B Biointerfaces 93, 36–40. 10.1016/j.colsurfb.2011.11.051 22244299

[B61] Robinson J. R. (1996). Introduction: semi-solid formulations of oral drug delivery. Bull. Tech. 11–14.

[B62] Ryu K. Lee M. K. Park J. Kim T. (2018). pH-responsive charge-conversional Poly(ethylene imine)–Poly(l-lysine)–Poly(l-glutamic acid) with self-assembly and endosome buffering ability for gene delivery systems. ACS Appl. Bio Mater. 1, 1496–1504. 10.1021/acsabm.8b00428 34996254

[B63] Saeed R. M. Dmour I. Taha M. O. (2020). Stable chitosan-based nanoparticles using polyphosphoric acid or hexametaphosphate for tandem ionotropic/covalent crosslinking and subsequent investigation as novel vehicles for drug delivery. Front. Bioeng. Biotechnol. 8:4. 10.3389/fbioe.2020.00004 32039190 PMC6993129

[B64] Sahay G. Querbes W. Alabi C. Eltoukhy A. Sarkar S. Zurenko C.. (2013). Efficiency of siRNA delivery by lipid nanoparticles is limited by endocytic recycling. Nat. Biotechnol. 31, 653–658. 10.1038/nbt.2614 23792629 PMC3814166

[B65] Sarmento B. Mazzaglia D. Bonferoni M. C. Neto A. P. do Céu Monteiro M. Seabra V. (2011). Effect of chitosan coating in overcoming the phagocytosis of insulin loaded solid lipid nanoparticles by mononuclear phagocyte system. Carbohydr. Polym. 84, 919–925. 10.1016/j.carbpol.2010.12.042

[B66] Schäfer J. Höbel S. Bakowsky U. Aigner A. (2010). Liposome–polyethylenimine complexes for enhanced DNA and siRNA delivery. Biomaterials 31, 6892–6900. 10.1016/j.biomaterials.2010.05.043 20561681

[B67] Senel G. Büyükköroglu G. Yazan Y. (2015). Solid lipid and chitosan particulate systems for delivery of siRNA. Pharmazie 70, 698–705. 10.1691/ph.2015.5026 26790185

[B68] Shahryari A. Jazi M. S. Mohammadi S. Nikoo H. R. Nazari Z. Hosseini E. S.. (2019). Development and clinical translation of approved gene therapy products for genetic disorders. Front. Genet. 10:868. 10.3389/fgene.2019.00868 31608113 PMC6773888

[B69] Shao X.-R. Wei X.-Q. Song X. Hao L.-Y. Cai X.-X. Zhang Z.-R.. (2015). Independent effect of polymeric nanoparticle zeta potential/surface charge, on their cytotoxicity and affinity to cells. Cell Prolif. 48, 465–474. 10.1111/cpr.12192 26017818 PMC6496505

[B70] Sharma D. Arora S. dos Santos Rodrigues B. Lakkadwala S. Banerjee A. Singh J. (2019). Chitosan-based systems for gene delivery, in Functional Chitosan: Drug Delivery and Biomedical Applications, eds. Jana S. Jana S. (Singapore: Springer Singapore), 229–267. 10.1007/978-981-15-0263-7_8

[B71] Shukla V. Seoane-Vazquez E. Fawaz S. Brown L. Rodriguez-Monguio R. (2019). The landscape of cellular and gene therapy products: authorization, discontinuations, and cost. Hum. Gene Ther. Clin. Dev. 30, 102–113. 10.1089/humc.2018.201 30968714

[B72] Sonvico F. Cagnani A. Rossi A. Motta S. Di Bari M. T. Cavatorta F.. (2006). Formation of self-organized nanoparticles by lecithin/chitosan ionic interaction. Int. J. Pharm. 324, 67–73. 10.1016/j.ijpharm.2006.06.036 16973314

[B73] Tariq I. Pinnapireddy S. R. Duse L. Ali M. Y. Ali S. Amin M. U.. (2019). Lipodendriplexes: a promising nanocarrier for enhanced gene delivery with minimal cytotoxicity. Eur. J. Pharm. Biopharm. 135, 72–82. 10.1016/j.ejpb.2018.12.013 30590107

[B74] Tezgel Ö. Szarpak-Jankowska A. Arnould A. Auzély-Velty R. Texier I. (2018). Chitosan-lipid nanoparticles (CS-LNPs): application to siRNA delivery. J. Colloid Interface Sci. 510, 45–56. 10.1016/j.jcis.2017.09.045 28934610

[B75] Thomas T. J. Tajmir-Riahi H. A. Pillai C. K. S. (2019). Biodegradable polymers for gene delivery. Molecules 24:3744. 10.3390/molecules24203744 PMC6832905 31627389

[B76] Trickler W. J. Munt D. J. Jain N. Joshi S. S. Dash A. K. (2011). Antitumor efficacy, tumor distribution and blood pharmacokinetics of chitosan/glyceryl-monooleate nanostructures containing paclitaxel. Nanomedicine 6, 437–448. 10.2217/nnm.10.135 21542683

[B77] Wagner M. Wiig H. (2015). Tumor interstitial fluid formation, characterization, and clinical implications. Front. Oncol. 5:115. 10.3389/fonc.2015.00115 26075182 PMC4443729

[B78] Wahane A. Waghmode A. Kapphahn A. Dhuri K. Gupta A. Bahal R. (2020). Role of lipid-based and polymer-based non-viral vectors in nucleic acid delivery for next-generation gene therapy. Molecules 25:2866. 10.3390/molecules25122866 32580326 PMC7356024

[B79] Whitehead K. A. Langer R. Anderson D. G. (2009). Knocking down barriers: advances in siRNA delivery. Nat. Rev. Drug Discov. 8, 129–138. 10.1038/nrd2742 19180106 PMC7097568

[B80] Wilhelm S. Tavares A. J. Dai Q. Ohta S. Audet J. Dvorak H. F.. (2016). Analysis of nanoparticle delivery to tumours. Nat. Rev. Mater. 1:16014. 10.1038/natrevmats.2016.14

[B81] Ziminska M. Wilson J. J. McErlean E. Dunne N. McCarthy H. O. (2020). Synthesis and evaluation of a thermoresponsive degradable chitosan-grafted PNIPAAm hydrogel as a “Smart” gene delivery system. Materials 13:2530. 10.3390/ma13112530 32498464 PMC7321466

