# Supplementary material for: Novel Ellipsoid Chitosan-Phthalate Lecithin Nanoparticles for siRNA Delivery
Source: Front Bioeng Biotechnol. 2021 Jul 28;9:695371. doi: 10.3389/fbioe.2021.695371 (PMC8355739; doi:10.3389/fbioe.2021.695371)
Supplement: Supplementary file 1 [file Data_Sheet_1.PDF]

Supplementary Information

**Sample Name:**	CP-L-TPP-siRNA 6		
**SOP Name:**	mansettings.nano		
**File Name:**	siRNA LOADED NPS.dts	**Dispersant Name:**	Water
**Record Number:**	6	**Dispersant RI:**	1.330
**Material RI:**	1.52	**Viscosity (cP):**	0.8872
**Material Absorbtion:**	0.000	**Measurement Date and Time:**	Monday, August 17, 2020 11:43:33 ...

**Temperature (°C):**	25.0	**Duration Used (s):**	50
**Count Rate (kcps):**	220.9	**Measurement Position (mm):**	4.65
**Cell Description:**	Disposable sizing cuvette	**Attenuator:**	7

			**Size (d.nm):**	**% Intensity:**	**St Dev (d.nm):**
**Z-Average (d.nm):**	214.1	**Peak 1:**	277.8	100.0	129.7
**PdI:**	0.225	**Peak 2:**	0.000	0.0	0.000
**Intercept:**	0.967	**Peak 3:**	0.000	0.0	0.000
**Result quality :**	Good				

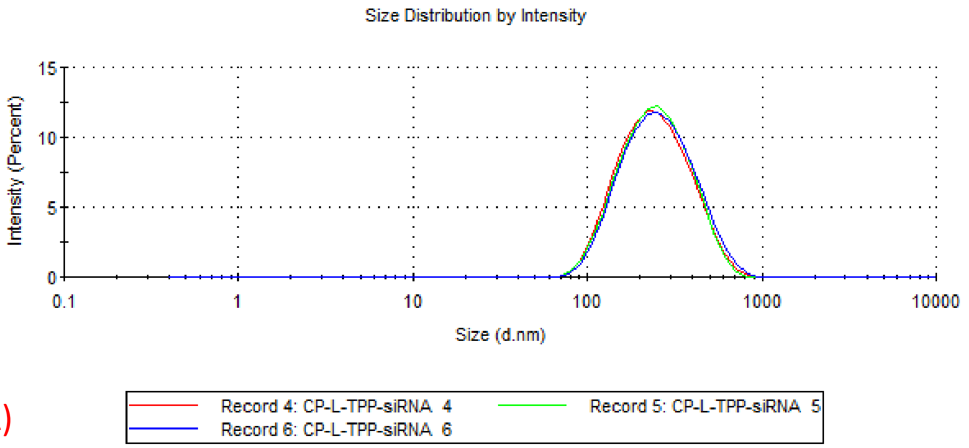

(A)

File Name:	C-L-PH and CaCL2 study 11082020.dts	Dispersant Name:	Water
Record Number:	231	Dispersant RI:	1.330
Date and Time:	Tuesday, August 11, 2020 1:53:49 PM	Viscosity (cP):	0.8872
		Dispersant Dielectric Constant:	78.5

Temperature (°C):	25.0	Zeta Runs:	12
Count Rate (kcps):	687.0	Measurement Position (mm):	2.00
Cell Description:	Clear disposable zeta cell	Attenuator:	9

			Mean (mV)	Area (%)	St Dev (mV)
Zeta Potential (mV):	30.0	Peak 1:	31.6	100.0	4.38
Zeta Deviation (mV):	22.6	Peak 2:	0.00	0.0	0.00
Conductivity (mS/cm):	1.01	Peak 3:	0.00	0.0	0.00

Result quality : Good

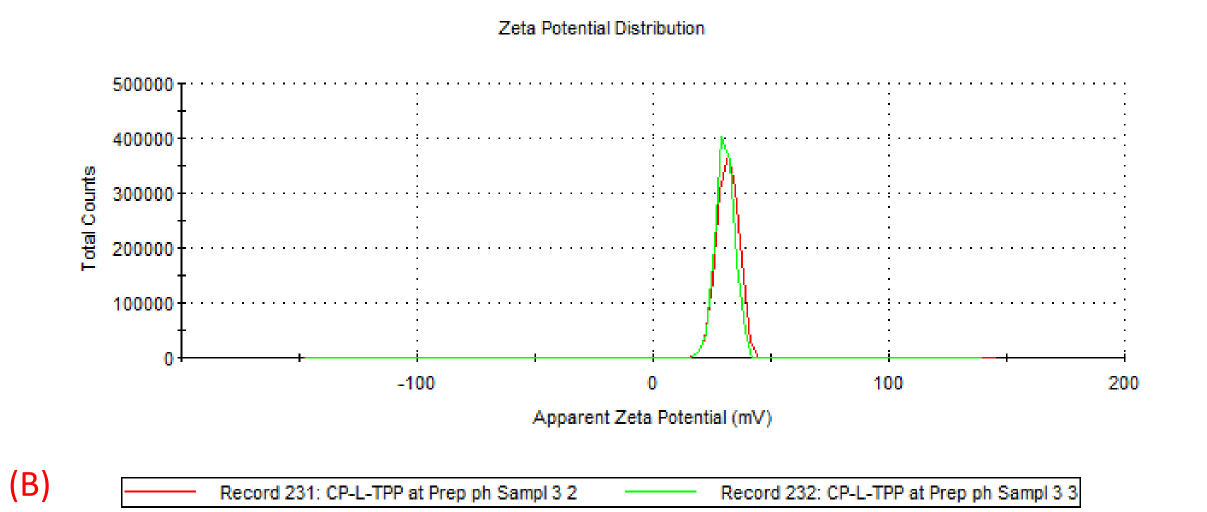

Figure S1. Dynamic light scattering parameters graph for (A) size distribution of CSP-LC-TPP NPs loaded with siRNA and (B) zeta potential of CSP-LC-TPP NPs.

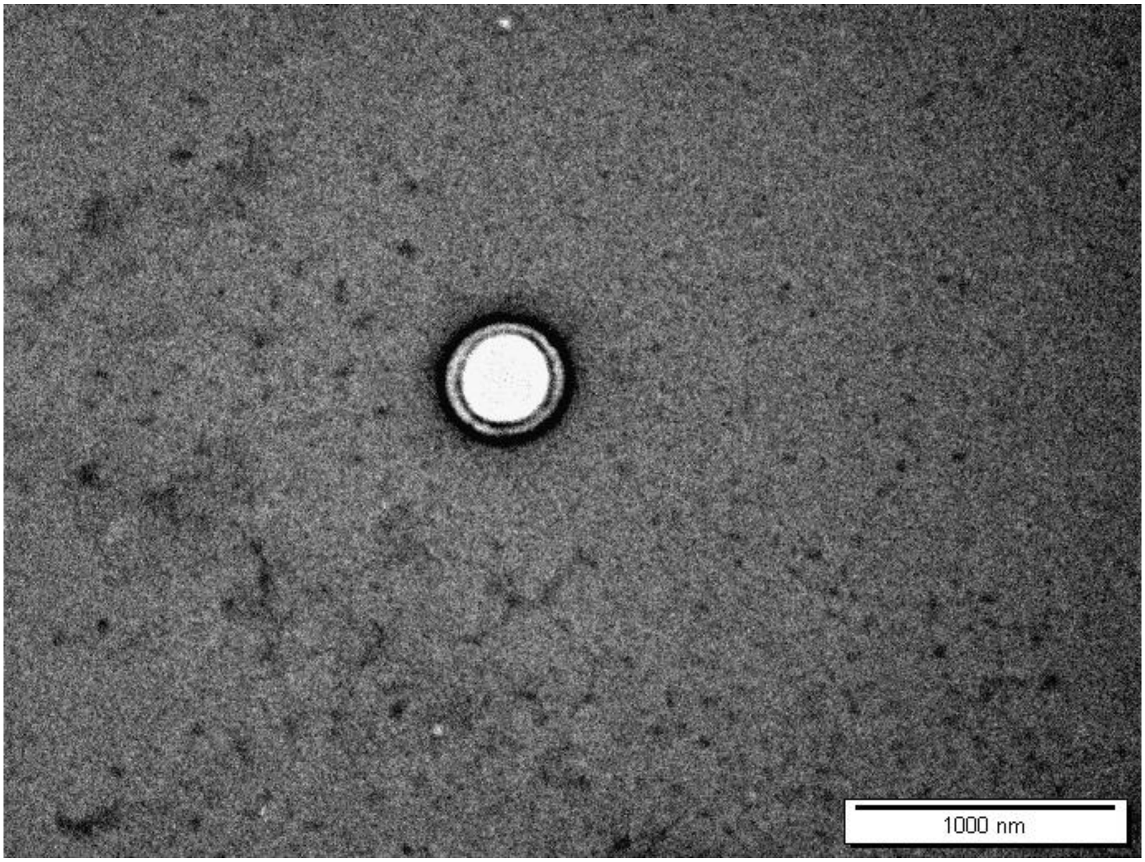

Figure S2. TEM image of TPP-crosslinked CS-LC NPs showing the double shell layers.

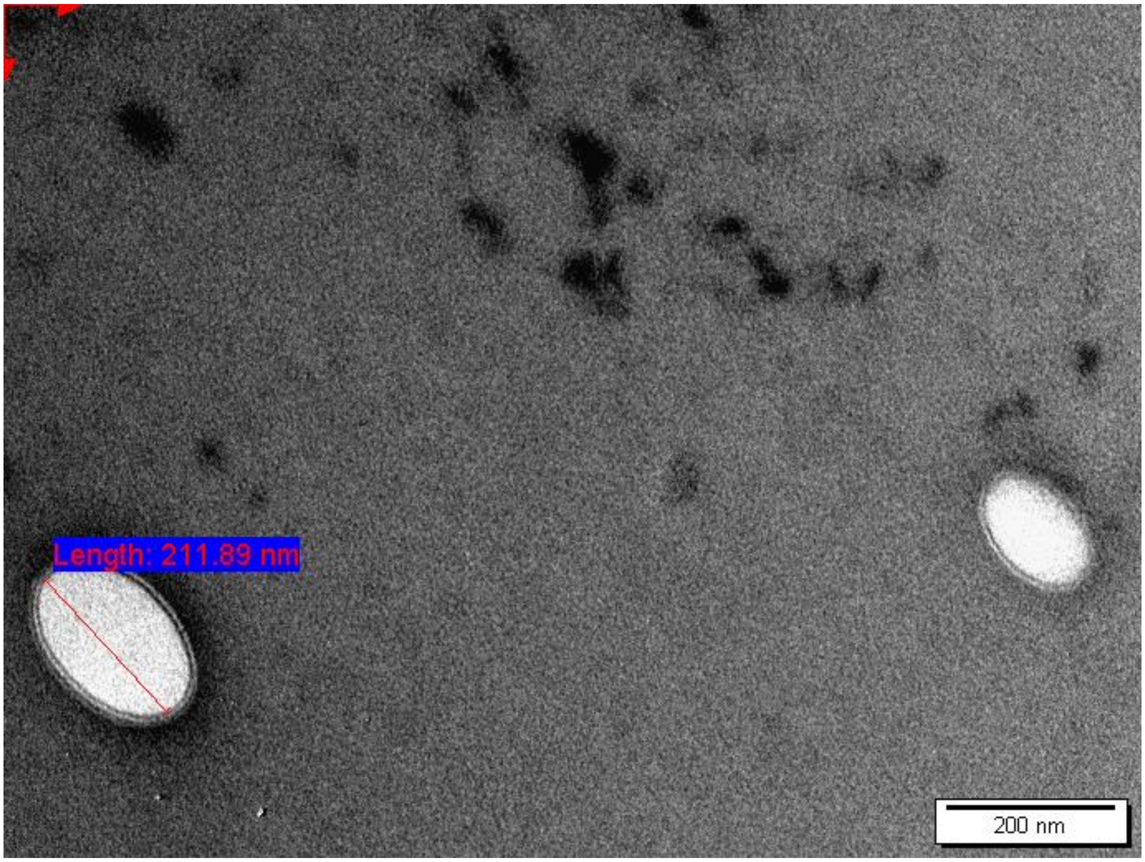

Figure S3. TEM image of TPP crosslinked CSP-LC NPs showing the double shell layers.

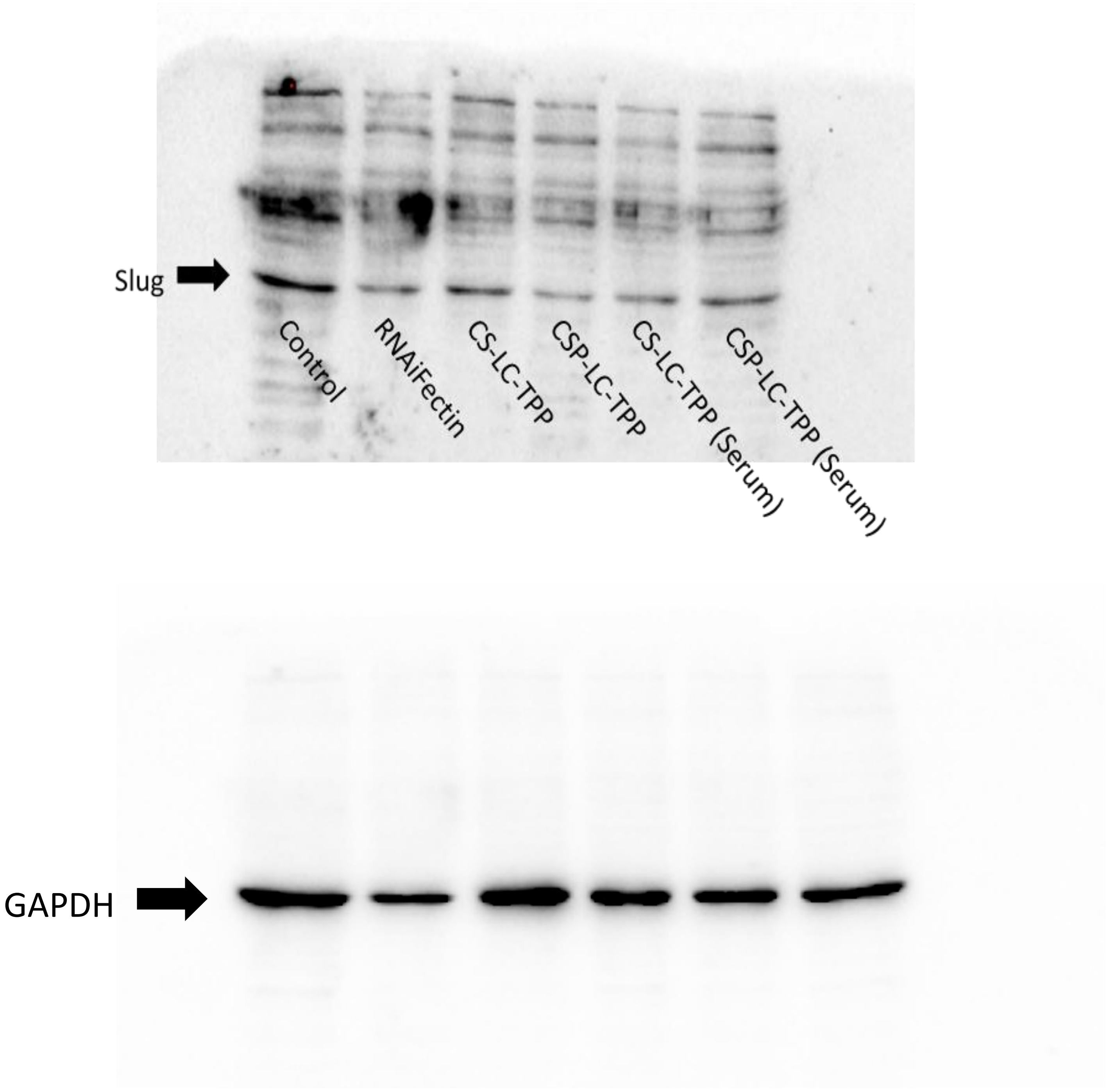

Figure S4a. Western blot membranes for single experiment for both SLUG and GAPDH proteins treated with Loaded NPs CS-LC-TPP, CSP-LC-TPP with and without serum. RNAiFectin was used as a positive control. Untreated cells were used as a control.

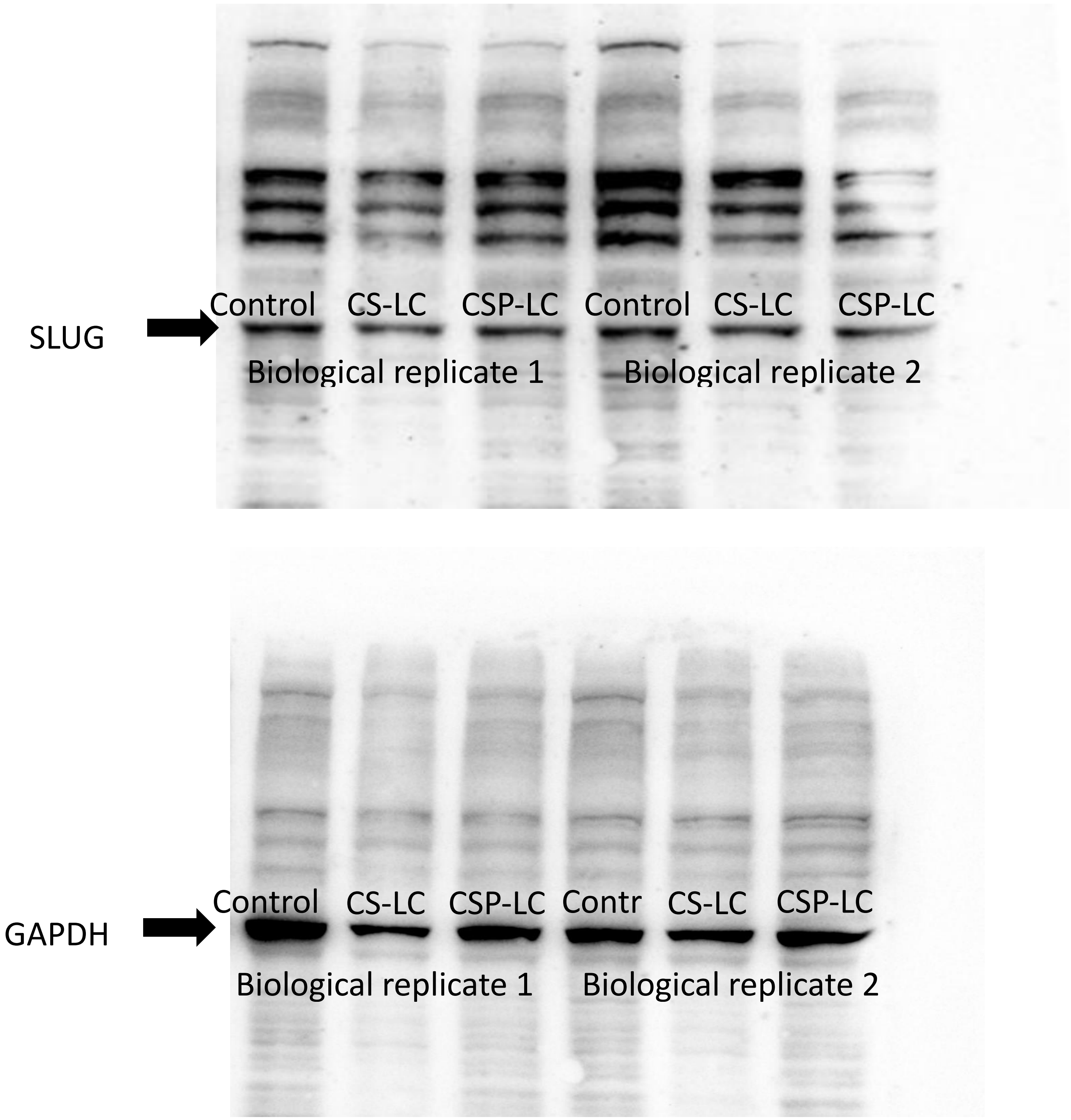

Figure S4b. Western blot membranes for single experiment for both SLUG and GAPDH proteins treated with Loaded NPs CS-LC, CSP-LC. Untreated cells were used as a control.

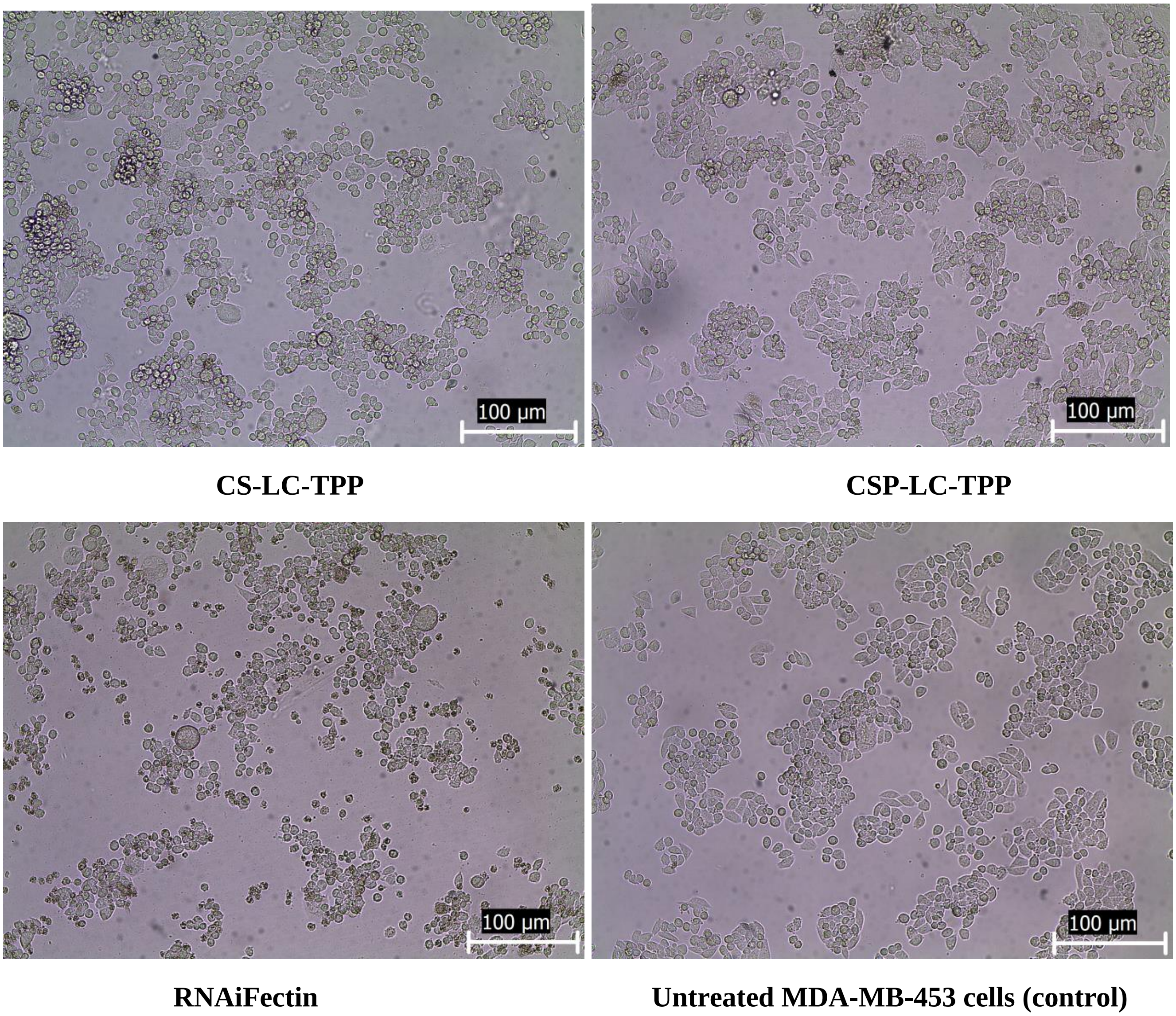

Figure S5. Light microscopy images for cell cytotoxicity associated with different blank NPs formulations compared to RNAiFectin. Untreated cell used as control.
